# Genetic Evolution of H9N2 Avian Influenza Virus in Guangxi, China

**DOI:** 10.3390/microorganisms13112579

**Published:** 2025-11-12

**Authors:** Minxiu Zhang, Sisi Luo, Zhixun Xie, Meng Li, Liji Xie, Qing Fan, Can Wang, Tingting Zeng, Hongyu Ren, Xiaofeng Li, Lijun Wan, Zhihua Ruan, Aiqiong Wu, Bingyi Yang, Houxun Ya, Ting-Rong Luo

**Affiliations:** 1 College of Animal Sciences and Veterinary Medicine, Guangxi University, Nanning 530004, China; zhminxiu2010@163.com; 2 Key Laboratory of China (Guangxi)-ASEAN Cross-Border Animal Disease Prevention and Control, Ministry of Agriculture and Rural Affairs, Guangxi Key Laboratory of Veterinary Biotechnology, Guangxi Veterinary Research Institute, 51 Youai North Road, Nanning 530001, China; luosisi2011@126.com (S.L.); mengli4836@163.com (M.L.); fanqing1224@126.com (Q.F.);

**Keywords:** H9N2 AIV, Guangxi, molecular evolution, genotype, gene sublineages

## Abstract

H9N2 avian influenza virus (AIV) is widely prevalent in poultry in China. To understand the genetic characteristics and evolution of H9N2 AIVs in Guangxi, southern China, the complete genomes of H9N2 AIVs from 1999–2023 were systematically analysed. Maximum likelihood (ML) trees indicated that H9N2 AIV gene sublineage diversity contributed to genotype diversity, yielding 17 genotypes (G1–G17). Since 2010, genotype G14 (also known as genotype S or G57) has become predominant in poultry in Guangxi. Phylogenetic analysis in the HA has resulted in the distancing of recent Guangxi isolates from the vaccine strains. This study also revealed that the genotypes of H9N2 AIVs infecting swine, equines and canines in Guangxi were consistent with those found in avian species at the same time, highlighting the capacity of H9N2 AIVs to be transmitted across species. The antigenic residues in the HA head region and NA protein of the Guangxi isolates from 2020–2023 changed significantly compared to the vaccine strains, suggesting possible antigenic drift in these viruses. Amino acid analysis of the HA protein revealed that 84.9% (73/86) of H9N2 AIV isolates from Guangxi, including those from live poultry markets, preferentially bound to α-2,6 sialic acid receptors. Considerable attention should be given to cross-species transmission of H9N2 AIV in the region. On the basis of these findings, strengthening the monitoring of H9N2 AIV in poultry in Guangxi is essential.

## 1. Introduction

H9N2 avian influenza virus (AIV) is very prevalent in wild birds and poultry worldwide. The first H9N2 AIV (A/Turkey/Wisconsin/1/1966) was isolated from a turkey in Wisconsin in the United States in 1966 [1]. In 1992, H9N2 AIV was first reported in mainland China, after which H9N2 AIV was gradually detected in live poultry in China [2]. Infection with a single H9N2 AIV strain does not cause poultry death; clinically, it manifests as depression, mild respiratory symptoms, and decreased egg production [3,4]. However, coinfection of H9N2 AIV with bacteria or viral pathogens can induce severe clinical outcomes, resulting in significant economic losses in poultry [5,6,7]. H9N2 AIV infects a broad range of hosts, including humans, wild birds, poultry, pigs and dogs. Furthermore, H9N2 AIV is a donor of the internal genes H7N9, H5N6, H10N8 and H3N8 AIV, which cause human infection; therefore, H9N2 AIV presents a significant risk to public health [8,9,10,11,12].

H9N2 AIVs are classified into North American and Eurasian lineages on the basis of phylogenetic analysis of HA genes [13]. The representative strain of the North American lineages is A/turkey/Wisconsin/1/1966. H9N2 AIVs of the North American lineage primarily circulate in wild birds but occasionally occur in turkeys [13]. H9N2 AIVs of Eurasian lineages are widely distributed among poultry and wild birds in regions such as Asia, Europe, and Africa [13]. H9N2 AIVs in China belong to the Eurasian lineage, which includes at least four sublineages: G1-like (represented by A/Quail/Hong Kong/G1/97), BJ/94-like (represented by A/chicken/Beijing/1/1994), Y439-like (represented by A/Duck/Hong Kong/Y439/97) and F/98-like (represented by A/Chicken/Shanghai/F/98) [14,15,16]. To further differentiate the evolutionary characteristics of the HA genes of H9N2 AIVs, Jiang divided H9N2 AIVs into four different evolutionary branches (9.1, 9.2, 9.3 and 9.4) according to phylogenetic analysis [17]. H9N2 AIVs in China are located mainly on the 9.4 evolutionary branch [17].

In 1998, the first inactivated H9N2 AIV vaccine (A/chicken/Shanghai/F/1998) was used in poultry to control the virus in China. Inactivated vaccines such as A/Chicken/Guangdong/SS/94 and A/Chicken/Shandong/6/96 were subsequently approved for marketing. After 1998, the majority of the poultry population in China were vaccinated with the inactivated H9 vaccine to control the spread of H9N2 AIV and reduce potential economic losses. Inactivated vaccines have significantly decreased the incidence of H9N2 AIV in poultry [4]. However, owing to the antigenic drift of the HA protein of H9N2 AIV, significant antigenic differences have emerged in the H9N2 AIV isolates within the predominant 9.4 evolutionary clade. Antigenic drift causes a mismatch between circulating strains and vaccine strains, which results in the current inactivated vaccines providing limited protection [18,19]. Sun et al. reported that even with high antibody levels following vaccination with the H9N2 AIV inactivated vaccine, the virus was still detected after challenge [20,21,22]. These findings indicate that H9N2 AIV infection remains common in immunized flocks. Therefore, monitoring and analysing the evolutionary characteristics of H9N2 AIVs is crucial for evaluating the compatibility of vaccine strains with prevalent strains, and this analysis provides valuable data for the timely updating of vaccines.

Guangxi, located in southern China, plays a significant role in livestock and poultry breeding, with approximately 1 billion poultry slaughtered annually. Monitoring data from live poultry markets (LPMs) in Guangxi since 2009 revealed that H9N2 AIV isolates accounted for 24.1% of the total low-pathogenicity avian influenza virus (LPAIV) isolates. This suggests a high prevalence of H9N2 AIV in LPMs in Guangxi [23], emphasizing the importance of remaining vigilant regarding the potential spillover risk of H9N2 AIV in LPMs. In this study, the genetic evolution of H9N2 AIV in Guangxi was systematically analysed by utilizing gene sequences from the GenBank database from 1999–2020 as well as H9N2 AIV isolates from LPMs from 2020–2023. These findings provide valuable data for the prevention and control of H9N2 AIV infection in Guangxi.

## 2. Materials and Methods

### 2.1. H9N2 AIV Isolates from 2020–2023

Twenty-seven H9N2 AIVs were isolated from LPMs in Guangxi from 2020–2023 in this study. Detailed information on these isolates is available in Supplementary Table S1. The protocols used for isolating and identifying these viruses have been described previously [23].

### 2.2. Full-Length Genome Sequencing and Assembly

The total viral RNA of the H9N2 AIV isolates was extracted following the instructions provided in the EasyPure Viral DNA/RNA Coextraction Kit (TransGen, Beijing, China). The viral RNA was reverse transcribed, and the full-length genome was amplified according to the protocols and primers described by Hoffmann [24]. The PCR products were subsequently purified using a gel extraction kit (Yisheng Biotechnology, Beijing, China). The target genes were subsequently cloned and inserted into the pMD18-T vector (TaKaRa, Dalian, China) for Sanger sequencing (Shenggong Bioengineering Co., Ltd., Shanghai, China). The full-length sequences were assembled by a company (Shenggong Bioengineering Co., Ltd., Shanghai, China).

### 2.3. Dataset Preparation

To analyse the epidemiological dynamics of H9N2 AIV in Guangxi, the gene sequences of 161 H9N2 AIV strains collected in the region from 1999–2020, along with human H9N2 AIV strains (n = 13) and domestic and foreign representative strains (n = 11) (Table S2), were downloaded from GenBank. Among the 161 H9N2 AIV isolates, the full-length genomes of 59 strains (the full-length genomes of Guangxi H9N2 AIV isolates from 2001 to 2004, 2015 to 2017 and 2019 are not available) were used for phylogenetic analysis in this study. The 59 isolates included 45 avian-derived H9N2 AIV strains, 9 swine-derived H9N2 AIV strains, 1 equine-derived H9N2 AIV strain, and 4 canine-derived H9N2 AIV strains (Table S2). The complete genomes of the 59 aforementioned isolates and 27 H9N2 AIV isolates from this study were imported into MEGA 7.0 software [25] on the basis of different genes (*PB2*, *PB1*, *PA*, *HA*, *NP*, *NA*, *M*, and *NS*) to generate distinct datasets, which were then exported in separate FASTA-formatted datasets. The MAFFT subroutine in BioAider v1.532 [26] was used to align these FASTA files. After alignment, the redundant regions at both ends were trimmed, and the results were exported in FASTA format. The FASTA format files were used to construct maximum likelihood (ML) trees.

### 2.4. Phylogenetic Analysis and Genotype Analysis

ML trees of the *PB2*, *PB1*, *PA*, *HA*, *NP*, *NA*, *M* and *NS* genes were constructed using IQ-TREE 2.0 software (UNIVIE, Vienna, Austria) following the methods described by Yang [27]. All the ML trees were constructed using the GTR + F + R4 model, with 1000 bootstrap replicates used to assess branch reliability. The ML trees were subsequently annotated using FigTree v1.4.3 software. The genotypes of H9N2 AIVs from Guangxi, collected between 1999 and 2023, were categorized based on phylogenetic analysis of eight genes (*PB2*, *PB1*, *PA*, *HA*, *NP*, *NA*, *M*, and *NS*) using the genotyping methods of H9N2 AIV described by Gu [28].

### 2.5. Classification of Sublineages

Gu classified the eight genes of H9N2 AIV into sublineages primarily on the basis of gene distance and phylogenetic tree topology. When H9N2 AIV isolates cluster with representative strains (A/Quail/Hong Kong/G1/97, A/chicken/Beijing/1/1994, A/Duck/Hong Kong/Y439/97, A/Chicken/Shanghai/F/98 and A/chicken/Hong Kong/G9/97), they are assigned to the corresponding G1-like, BJ/94-like, Y439-like, F/98-like and G9/97-like sublineages, respectively [28]. Jiang classified the HA gene evolutionary clade of the BJ/94-like sublineage on the basis of genetic distance and phylogenetic tree topology [17]. The evolutionary clades within the BJ/94-like sublineage can be classified into six evolutionary clades. The clades are represented as follows: h9.4.2.1 by A/chicken/Henan/2/98, h9.4.2.2 by A/wild duck/Shantou/4808/01, h9.4.2.3 by A/chicken/Hong Kong/G9/97, h9.4.2.4 by A/chicken/Hong Kong/Y280/97, h9.4.2.5 by A/chicken/Guangxi/55/2005, and h9.4.2.6 by A/chicken/Guangdong/FZH/2011 [17].

### 2.6. Classification of Genotypes

The sublineages of the eight genes of H9N2 AIV were clarified on the basis of phylogenetic analysis. According to a study by Gu, eight genes from different sublineages of H9N2 AIV were reassorted to form diverse genotypes [28]. In this study, the genotypes of H9N2 AIVs in Guangxi were classified using Gu’s method [28]. The genotypes in this study are denoted as “G + number”. Genotype IDs were assigned according to the isolation time of the initial founder isolate.

### 2.7. Amino Acid Analysis

The differences in the amino acid residues of the HA and NA proteins between H9N2 AIV from Guangxi (1999 to 2023) and classical vaccine strains used in China (A/chicken/Shanghai/F/1998, A/chicken/Guangdong/SS/94, and A/chicken/Handong/6/96) were analysed in this study, with a focus on amino acid residues in the HA head region and NA protein recognized as contributing to antigenic drift [29,30,31,32,33,34,35,36,37,38,39,40]. Additionally, the homology of the HA protein was analysed using BioAider v1.532 to compare H9N2 AIVs from Guangxi (1999 to 2023) with vaccine strains [26]. The key amino acid sites of the HA, NA, PB2, and M2 proteins were analysed using MEGA 7.0 software [25]. These critical sites include antigenic sites in the HA head region, the cleavage site and the receptor-binding site; the stem deletion amino acid sites and resistance sites in the NA protein; the sites associated with interspecies transmission and mammalian pathogenicity in the PB2 protein; and the resistance sites in the M2 protein.

## 3. Results

### 3.1. Prevalence of H9N2 AIV Infection in Guangxi from 1999–2023

**Figure 1:**
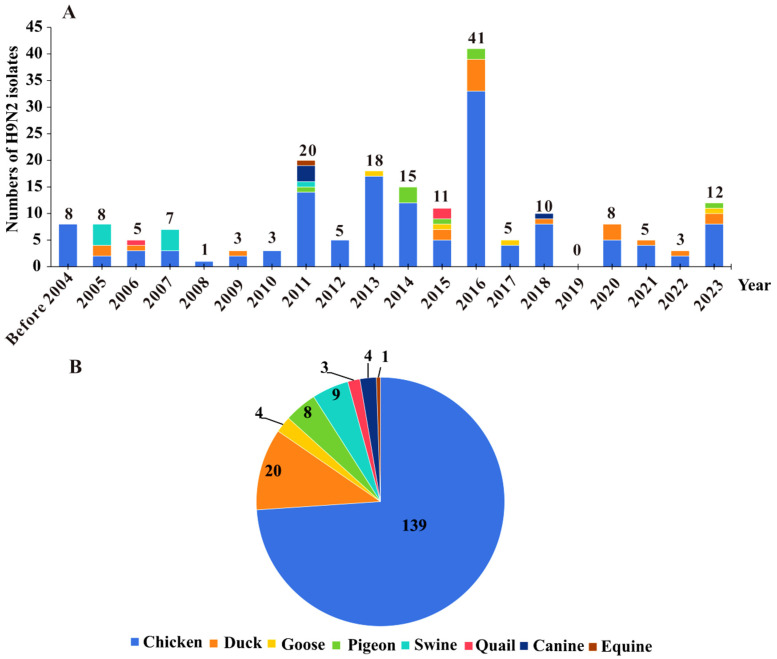
Numbers of H9N2 AIV isolates from different years and different host species in Guangxi. (**A**) Numbers of H9N2 AIV isolates in different years in Guangxi; (**B**) Numbers of H9N2 AIV isolates among different host species in Guangxi.

A total of 161 H9N2 AIV isolates from poultry, equine, swine, and canine samples in Guangxi were obtained from the GenBank database between 1999 and 2020. These 161 H9N2 AIV isolates were combined with the 27 H9N2 AIV strains obtained in this study to form a dataset. The dataset was statistically analysed on the basis of collection date, host species, and number of isolates. The results revealed that the majority of the isolates (n = 125) were collected between 2011 and 2018, with a peak occurring in 2016, when 41 strains were identified (Figure 1A). The distribution of H9N2 AIVs in Guangxi across different host species revealed a broad range of susceptible hosts, with chickens being the primary host at 73.9%, followed by ducks (10.6%), swine (4.8%), pigeons (4.3%), canines (2.1%), geese (2.1%), quail (1.6%), and equines (0.5%) (Figure 1B).

### 3.2. Genetic Evolutionary Analysis of H9N2 AIV Isolates in Guangxi

**Figure 2:**
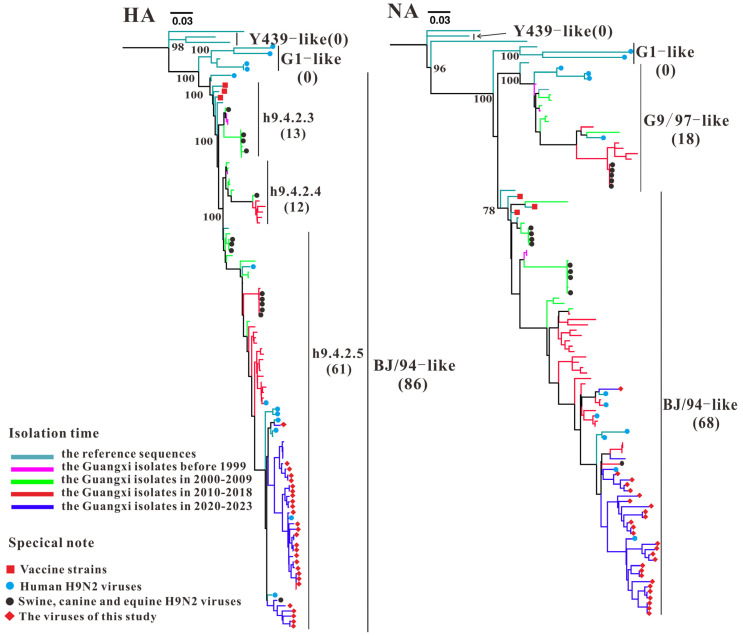
Phylogenetic analysis of the *HA* and *NA* genes of H9N2 AIV. The numbers in parentheses represent the number of Guangxi H9N2 AIV strains. Note: The phylogenetic trees, including strain names, is presented in Figure S1.

**Figure 3:**
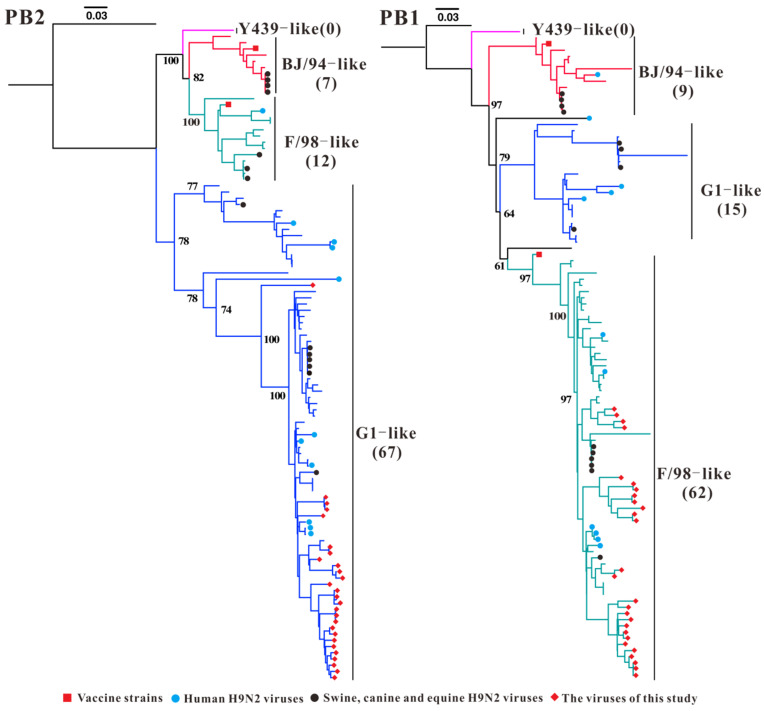
Phylogenetic analysis of the *PB2* and *PB1* genes of H9N2 AIV. Different colour branches represent different sublineages; the numbers in parentheses represent the number of Guangxi H9N2 AIV strains. Note: The phylogenetic trees, including strain names, is presented in Figure S1.

**Figure 4:**
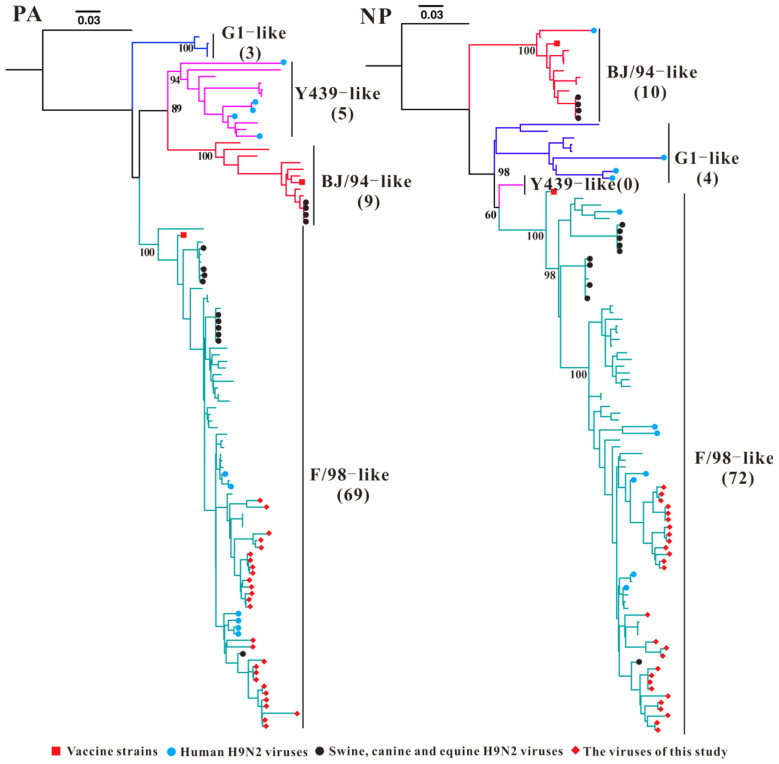
Phylogenetic analysis of the *PA* and *NP* genes of H9N2 AIV. Different colour branches represent different sublineages; the numbers in parentheses represent the number of Guangxi H9N2 AIV strains. Note: The phylogenetic trees, including strain names, is presented in Figure S1.

**Figure 5:**
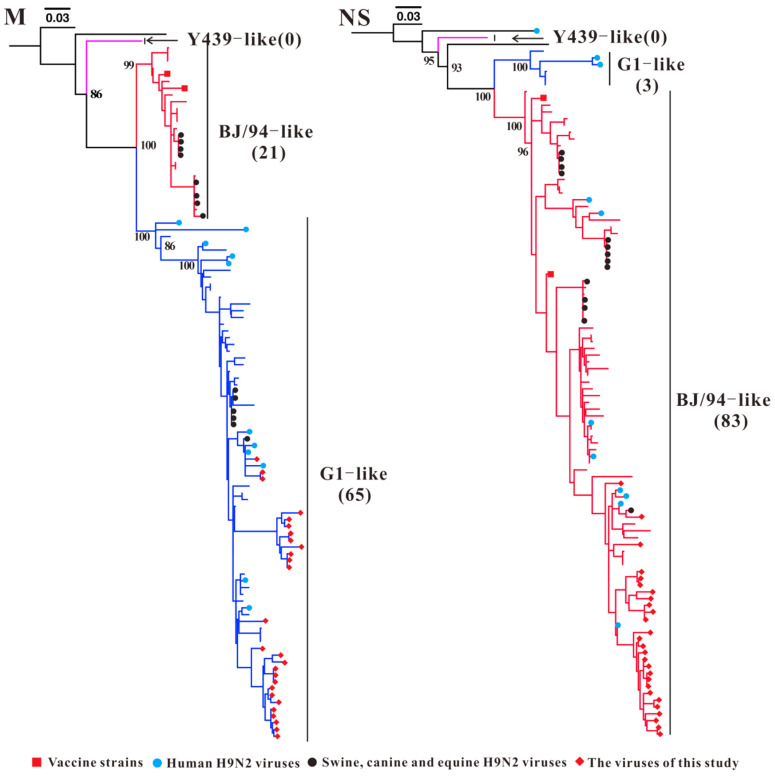
Phylogenetic analysis of the *M* and *NS* genes of H9N2 AIV. Different colour branches represent different sublineages; the numbers in parentheses represent the number of Guangxi H9N2 AIV strains. Note: The phylogenetic trees, including strain names, is presented in Figure S1.

ML trees of the *PB2*, *PB1*, *PA*, *HA*, *NP*, *NA*, *M*, and *NS* genes were constructed using 59 Guangxi H9N2 AIV isolates from GenBank (1999–2020), 27 H9N2 AIV isolates from this study (2020–2023), 13 human H9N2 AIV isolates, and 11 domestic and foreign representative isolates. The results are shown in Figure 2, Figure 3, Figure 4 and Figure 5. All Guangxi H9N2 AIV isolates (n = 86) from 1999–2023 belonged to the BJ/94-like sublineage according to the ML tree of *HA* genes (Figure 2 and Figure S1). In accordance with the classification of the evolutionary clade of the BJ/94-like sublineage described by Jiang [17], the Guangxi H9N2 AIV isolates of the BJ/94-like sublineage can be further divided into h9.4.2.3 (n = 13), h9.4.2.4 (n = 12), and h9.4.2.5 (n = 61), with h9.4.2.5 being the dominant clade that is currently circulating in China. The ML tree based on the *NA* genes revealed that Guangxi H9N2 AIVs can be divided into G9/97-like (n = 18) and BJ/9-like (n = 68) sublineages (Figure 2 and Figure S1). The evolutionary clades of the *HA* and *NA* genes of the Guangxi isolates in the BJ/94-like lineage exhibited distinct distribution characteristics over time. In addition, the strains isolated in this study are relatively distantly related to vaccine strains (A/chicken/Shanghai/F/1998, A/chicken/Guangdong/SS/94, and A/chicken/Handong/6/96) (Figure 2).

The sublineages of the *PB2*, *PB1*, *PA* and *NP* genes of H9N2 AIV isolated from Guangxi from 1999–2023 exhibited diverse characteristics (Figure 3 and Figure 4 and Figure S1). On the basis of the *PB2* phylogenetic tree, a total of 77.9% (67/86) of the Guangxi isolates belonged to the G1-like sublineage, including 58 strains isolated from 2010–2023 as well as 10 human-derived H9N2 AIV strains (excluding A/Hong_Kong/3239/2008) (Figure 3 and Figure S1). The genetic evolution of the *PB1* genes of the Guangxi H9N2 AIVs revealed that 72.1% (62/86) of the Guangxi isolates belonged to the F/98-like sublineage. The Guangxi H9N2 AIV isolate containing the F/98-like sublineage *PB1* was first recorded in 2006, after which the virus with the F/98-like sublineage *PB1* tended to stabilize.

Phylogenetic analysis of the *PA* gene from Guangxi H9N2 AIVs revealed that the *PA* gene of H9N2 AIVs can be divided into four sublineages: BJ/94-like (n = 9), G1-like (n = 3), F/98-like (n = 69), and Y439-like (n = 5) (Figure 4). The *NP* genes of Guangxi H9N2 AIVs were divided into those of the BJ/94-like (n = 10), F/98-like (n = 72), and G1-like (n = 4) sublineages. The earliest isolates with F/98-like sublineages for *PA* and *NP* were reported in 2005. The *PA* and *NP* genes of Guangxi isolates collected from 2010–2023 formed a large F/98-like clade (Figure 4 and Figure S1).

The classification of the sublineages of the *M* and *NS* genes of H9N2 AIVs from Guangxi was relatively straightforward (Figure 5). H9N2 AIVs from Guangxi were divided into BJ/94-like (n = 21) and G1-like (n = 65) sublineages on the basis of the *M* gene phylogenetic tree. The *NS* genes of H9N2 Guangxi AIVs were also divided into those of the BJ/94-like (n = 83) and G1-like (n = 3) sublineages (Figure 5 and Figure S1). The earliest Guangxi isolate, including the G1-like sublineage of the *M* gene, was isolated in 2005, and all Guangxi H9N2 AIVs isolated after 2008–2023 and human-derived H9N2 AIVs (n = 11) were in the G1-like lineage (Figure S1). The *NS* genes of 83 Guangxi isolates collected from 1999–2023 formed a large BJ/94-like clade, indicating that the genetic evolution of H9N2 AIV in the BJ/94-like sublineage of the *NS* gene has been very stable.

In this study, the internal genes of Guangxi H9N2 AIVs isolated from 2010–2023 included *PB2* and *M* genes from the G1-like sublineage; *PB1*, *PA*, and *NP* genes from the F/98-like sublineage; and *NS* genes from the BJ/94-like sublineage. The earliest record for Guangxi H9N2 isolates containing F/98-like sublineage *PB1*, *PA*, and *NP* genes and G1-like lineage *M* genes dates from 2005–2006. However, owing to the absence of isolates from 2001–2004, the introduction of viruses with F/98-like and G1-like sublineages into poultry may have occurred prior to 2005.

### 3.3. Genotype Analysis

**Figure 6:**
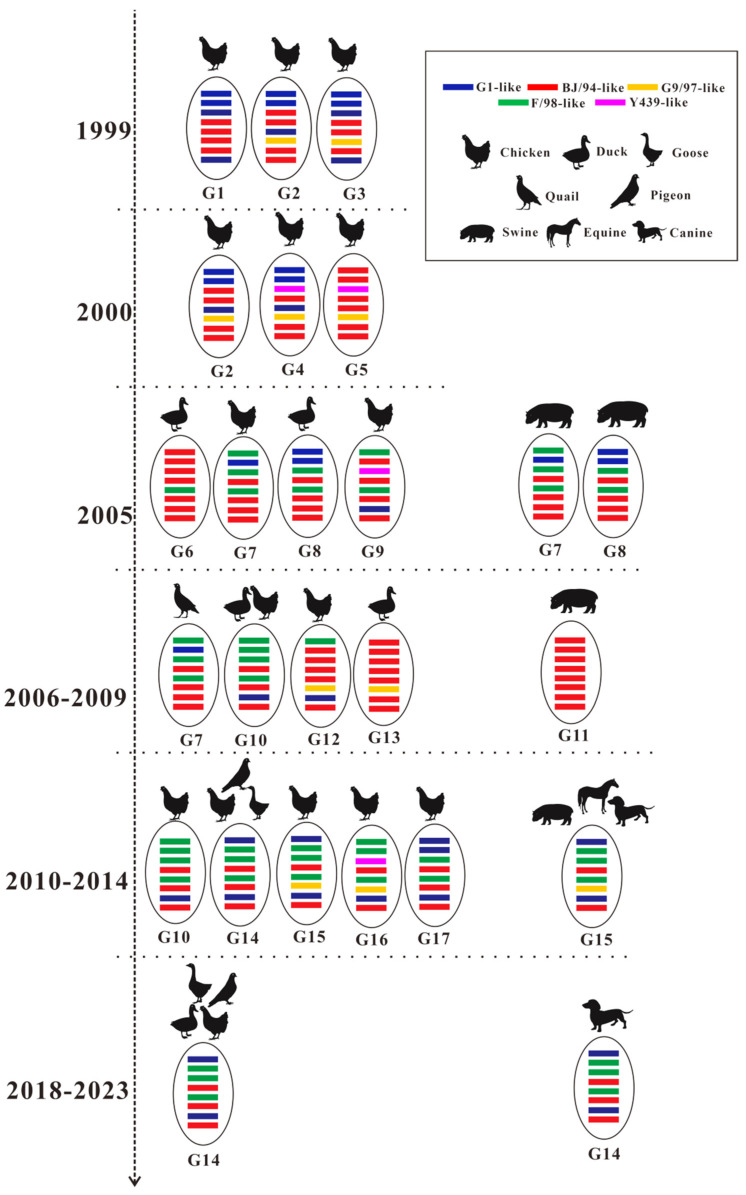
Evolution of H9N2 AIV genotypes in Guangxi from 1999–2023. The eight horizontal bars in the oval (from top to bottom) represent the *PB2*, *PB1*, *PA*, *HA*, *NP*, *NA*, *M*, and *NS* genes. Each colour corresponds to a specific virus lineage.

In a previous study, Gu classified H9N2 AIVs isolated in China from 1994–2014 into 23 genotypes (A-W) [28]. Genotype analysis of H9N2 AIVs isolated in Guangxi from 1999–2023 revealed 17 genotypes (G1–G17) (Figure S2). G1, G3, G10 G11, G13, G14, and G15 were consistent with genotypes D, C, O, A, F, S, and V, respectively, as described by Gu [28]. However, the other 16 genotypes (B, E, G, H, I, J, K, L, M, N, P, Q, R, T, U, and W) were not found in Guangxi H9N2 AIVs, and new genotypes, G2, G4-G9, G12, and G16-G17, were identified. Since genotype G14 appeared in poultry in Guangxi in 2010, genotype G14 (also known as genotype S or genotype G57 in China) has been able to spread stably among poultry in Guangxi, and this genotype has also been isolated from canines in the region (Figure 6 and Figure S2).

**Figure 7:**
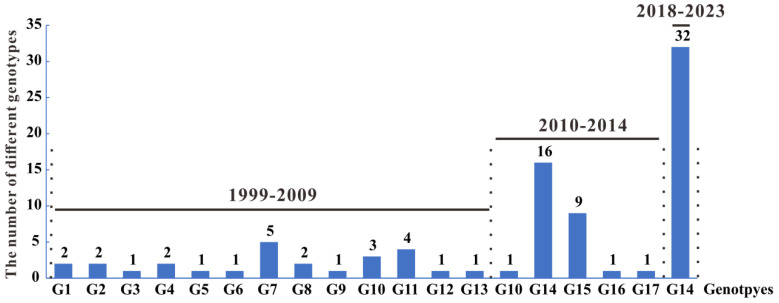
The number of H9N2 AIV isolates of the genotypes at different times in Guangxi. Different time periods are separated by dashed lines.

The genotypes infecting different hosts during different periods in Guangxi were further analysed (Figure 6 and Figure 7). A total of 13 genotypes (G1–G13) were identified from 1999–2009, with no dominant genotype observed. Five genotypes (G10 and G14–G17) were identified in poultry between 2010 and 2014, with G14 being the dominant genotype from 2010–2014 in poultry, whereas the genotype of the swine-, equine-, and canine-derived H9N2 AIVs was G15. Only one genotype (G14) was identified among poultry-derived H9N2 AIVs from 2018–2023, and the genotype of an H9N2 AIV from a canine was also G14 (Figure 6). These results indicate that the genes of H9N2 AIVs in Guangxi frequently underwent genetic reassortment in poultry before 2009, leading to the formation of different genotypes; however, no dominant genotype was established. After 2010, G14 spread widely among poultry in Guangxi and gradually became the dominant genotype. This genotype stabilized in the region’s poultry industry after 2018. Additionally, the genotypes identified in both poultry and mammals during the same period were identical (except for genotype G11), suggesting that the same genotype of H9N2 AIV infection in poultry may have spread to mammals.

### 3.4. Amino Acid Analysis of H9N2 AIVs in Guangxi

#### 3.4.1. Molecular Analysis of the HA Protein

Homology analysis of the HA protein was performed between Guangxi H9N2 AIVs isolated during different periods and three vaccine strains (A/chicken/Shanghai/F/1998, A/chicken/Guangdong/SS/94, and A/chicken/Shandong/6/96). Amino acid identities between the Guangxi H9N2 AIVs and the vaccine strains ranged from 85.9 to 95.38% from 1999 to 2010, 80.46 to 88.63% for isolates from 2010 to 2018, and 77.80% to 81.88% for isolates from 2020 to 2023. Amino acid analysis of the HA protein was performed between the Guangxi H9N2 AIVs and the vaccine strains on the basis of the 30 antigenic sites in the HA head region as previously reported to evaluate antigenic differences at the molecular level [29,30,31,32,33,34,35,36,37,38] (Tables S3 and S4). Analysis of the antigenic sites on the HA head region of H9N2 AIVs from 1999 to 2023 revealed that ten amino acid residues (Q133, T138, T147, P170, T206, D207, E215, L230, R252, and K282) were relatively conserved; except for position 282, the other nine amino acid residues were identical to those of the vaccine strains. In comparison to those in the three vaccine strains, the 18 antigenic residues in the HA head region of the Guangxi isolates from 2020 to 2023 changed significantly, including G90E, S145D, K149N, D153G, T163N, Q164R, N167G, A168N, R180Q, D196E, T197D, T200R, N201T, T220I, Q234L, Q235M, V287T, and R294K. The proportions of 90E, 145D, 149N, 153G, 163N, 164R, 167G, 168N, 180Q, 196E, 197D, 200R, 201T, 220I, 234L, 235M, 287T, and 294K increased in the isolates from 2011 to 2023 (Table S3). The HA asparagine (Asn, N) → aspartic acid (Asp, D) mutation at position 166 has been demonstrated to enhance immune escape in H9N2 AIVs [36,41]. The prevalence of the Asn166 strain in the HA protein of Guangxi isolates was 68.6% (59/86) (Table S5). The Ck/GX/418C4/20, Du/GX/422D33/20, Ck/GX/LZ076D7/20, Ck/GX/LZ076E7/20, Ck/GX/472C2/21, and Ck/GX/472C4/21 isolates in this study presented Asp at position 166, indicating the potential for immune escape (Table S5). These mutations in the HA protein play important roles in the emergence of immune escape variants.

Amino acid analysis of the HA protein cleavage site indicated that H9N2 AIV isolates in Guangxi are low-pathogenicity isolates (Table S5). Further analysis revealed that the cleavage motif in the HA proteins of H9N2 AIV isolates from Guangxi after 2014 was PSRSSR ↓ GL (Table S5), indicating that the isolates containing PSRSSR↓GL cleavage motifs were predominant in poultry from Guangxi.

The critical amino acid site of the HA protein that affects the receptor binding specificity and affinity of the H9N2 AIV for avian (α-2,3 sialic acid receptors) or human sialic acid receptors (α-2,6 sialic acid receptors) has been identified at positions 234, 198 and 235 (H9 numbering) [42,43,44]. In this study, 84.9% (73/86) of the HA proteins of Guangxi H9N2 AIVs at position 234 were isoleucine (Ile, L) (Table 1 and Table S6), indicating that these viruses preferentially bind to the α-2,6 sialic acid receptors. The HA proteins of 27 Guangxi H9N2 AIVs displayed 234L and 198V, suggesting that these viruses have increased affinity for the human sialic acid receptor (Table S5) [43]. The combinations of amino acid residues at positions 234 and 235 of the HA protein affect receptor affinity, replication and transmission in chickens. The number of isolates with amino acid combinations at positions 234L and 235M gradually increased in Guangxi after 2013 (Tables S6 and S7). A total of 93.3% (42/45) of the Guangxi isolates isolated after 2013 were classified as LM isolates, indicating that these strains of H9N2 AIVs predominated in the poultry of Guangxi from 2013–2023 (Table S7). These results suggest that the isolates with HA protein positions 234 and 235 as LM have an advantage in terms of transmission among poultry.

#### 3.4.2. Molecular Analysis of the NA, PB2 and M2 Proteins

Among the 68 isolates in the BJ/94-like sublineage based on the NA ML tree, 63 presented deletions of amino acids at positions 63–65 in the NA protein, which is beneficial for virus adaptation to terrestrial poultry (Table 1 and Table S5) [45,46]. The results also revealed that all Guangxi isolates from the G9/97-like sublineages did not display these deletions in NA proteins. Position 274 in the NA protein of the Guangxi isolates was a histidine (His, H), which suggested that all the Guangxi isolates were sensitive to neuraminidase inhibitors (Table 1 and Table S5). Amino acid analysis of the NA protein was performed between the Guangxi H9N2 AIVs and the vaccine strains on the basis of the 12 antigenic sites in the NA protein as previously reported (Tables S4 and S8) [39,40]. Analysis of the antigenic sites on the NA protein of H9N2 AIVs from 1999 to 2023 revealed that four amino acid residues (D198, G248, R253 and S400) were conserved. In comparison to those in the three vaccine strains, the 5 antigenic residues in the NA protein of the Guangxi isolates from 2020 to 2023 changed significantly, including G125S, R344K, N356D, K/E368N, and D369G/S. The proportions of 125S, 344K, 356D, 368N, and 369G/S increased in the isolates from 2011 to 2023 (Table S8).

The amino acid residues at positions 627 and 701 in the PB2 protein of the Guangxi isolates were glutamic acid (Glu, E) and Asp, respectively, which suggested that these viruses exhibited weak pathogenicity in mice (Table 1 and Table S5). A total of 73.2% (63/86) of the Guangxi isolates displayed an Asn at position 31 of the M2 protein, indicating that these viruses may be insensitive to amantadine (Table 1 and Table S5).

**Table 1:** Amino acid substitutions in H9N2 AIVs contribute to enhanced pathogenicity, receptor binding, and transmissibility in mammals.

Protein	Site	Amino Acids (No.)	Possible Function
HA (H9 Numbering)	333–338	PARASR(6), PARSSR (19), PSKSSR (1), PSRSSR (60)	Two discontinuous basic amino acids are a typical feature of low-pathogenicity avian influenza
	234	Q (13), L (73)	The 234L mutation enables the virus to preferentially bind to α-2,6 sialic acid receptors [42]
	198	A (25), T (32), V (29)	The 234L and 198V mutations increase affinity for the α-2,6 sialic acid receptors and promote viral replication in mouse or human lung tissues [43]
	235	Q (42), M (43), L (1)	The 234L and 235M mutations show a distinct advantage in replication and transmission within the chicken infection group [44]
	166	D (26), N (59), T (1)	166D enhances immune escape [36,41]
NA (H9 Numbering)	63–65	Stalk deletion (63), No stalk deletion (23)	Stalk deletion at the NA protein increases replication and virulence in chickens and mice [47]
	274	H (86)	274H exhibits sensitivity to neuraminidase inhibitors [48]
PB2	627	E (86)	The 627E and 701D mutations exhibit weak pathogenicity in mice [49,50,51]
	701	D (86)	
M2	31	S (22), N (63), G (1)	S31N provides viral resistance to amantadine [52,53]

## 4. Discussion

H9N2 AIV is the predominant subtype of AIV and is widely prevalent in poultry in China [17]. Wang reported a significant increase in the isolation rate of H9N2 AIV in 2016 while monitoring this virus in poultry in eastern China (specifically in Jiangsu, Anhui, Shandong, and Zhejiang Provinces) from 2010–2020. This increase may be associated with the occurrence of H9N2 avian influenza in chicken flocks [54]. The number of H9N2 AIV isolates in Guangxi from 1999–2023 was analysed in this study. The results revealed that the greatest number of sequences was recorded in 2016, which is consistent with the findings of Wang, suggesting that H9N2 AIV may have undergone regional circulation in poultry in Guangxi in 2016.

A vaccination strategy for controlling H9N2 AIV in China has been implemented. However, long-term vaccination may accelerate the evolution of H9N2 AIV. The antigenic changes in H9N2 AIVs are caused primarily by mutations in the amino acid sites of HA proteins, which enable the virus to escape immune responses [55]. Previous studies have demonstrated that the isolates in the predominant clade (h9.4.2.5) of H9N2 AIVs in China can be divided into at least three antigen groups according to antigenic differences, resulting in the vaccine providing limited protection for specific pathogen-free chickens [18,19,41]. Studies have also confirmed that the antigenicity of different antigen groups is significantly correlated with their isolation date [19,41]. Phylogenetic analysis of the *HA* gene of H9N2 AIVs indicated that the Guangxi H9N2 AIV isolates primarily belong to the h9.4.2.5 clade of the BJ/94-like lineage. These isolates exhibit distinct temporal distribution characteristics, which suggests the possibility of different antigen groups in Guangxi. In addition, all 27 isolates obtained from the LPMs from 2020–2023 in this study were relatively distant from the vaccine strains, which indicated that continuous variation in the HA protein may have resulted in antigenic changes in H9N2 AIVs in this region in recent years.

Changes in the antigenicity of H9N2 AIVs are closely associated with mutations in the HA head antigen epitopes. Multiple studies have identified antigenic sites on the HA head region, and mutations at these sites can help the virus escape the immune system by altering receptor binding properties, masking through glycosylation, and altering antibody-binding regions. Zhang reported that simultaneous mutations at eight antigenic sites (G90E, S145D, K149N, D153G, Q164R, N166D, N167G, and A168N) can result in changes in viral antigenicity [56]. The N201T mutation in the HA head region may cause the emergence of antibody escape mutants [31]. Zheng reported that co-mutations at positions 145D, 153G, 163N, and 164R can cause antigenic drift and immune escape [35]. In this study, the antigenic sites in the HA head region of Guangxi H9N2 AIVs were compared with those of classical vaccine strains. The HA head region of Guangxi H9N2 AIVs from 2011–2023, especially the isolates from 2020–2023, showed mutations at the following positions: G90E, S145D, K149N, D153G, T163N, Q164R, N167G, A168N, R180Q, D196E, T197D, T200R, N201T, T220I, Q234L, Q235M, V287T, and R294K. Molecular analysis revealed that the Guangxi H9N2 AIVs from 2020–2023 exhibited significant changes at antigenic sites in HA protein. In addition to mutations at key antigenic sites of HA protein, the NA protein of Guangxi isolates from 2020–2023 also exhibited mutations (R344K, N356D, and D369G/S) at critical antigenic sites. Previous studies have demonstrated that these mutations can directly disrupt antibody-binding epitopes or alter the spatial conformation of the NA head region, which may reduce antibody binding and inhibition activity, NA antigenic change might have occurred in the Guangxi H9N2 viruses [40]. The changes in the antigenic sites of Guangxi H9N2 AIVs may cause antigenic drift, contributing to immune failure; however, further verification is needed to confirm this hypothesis.

H9N2 AIVs have been confirmed to form different genotypes through genetic reassortment. According to the results of the phylogenetic and genotypic analyses in this study, the predominant sublineage of the *HA*, *NA*, and *NS* genes in H9N2 AIV in Guangxi from 1999–2023 was the BJ/94-like sublineage. At least three sublineages (G1-like, BJ/94-like, Y439-like or F/98-like) of the *PB1*, *PA*, and *NP* genes were involved in the evolution and reassortment of the virus between 1999 and 2009 in Guangxi. However, with the introduction of H9N2 AIVs from A/Chicken/Shanghai/F/98 origin in poultry in Guangxi, H9N2 AIVs containing *PB1*, *PA*, and *NP* genes from the F/98-like sublineage replaced other lineages after 2010. The evolutionary trajectory of the PB2 sublineage differed from that of the *PB1*, *PA*, and *NP* genes. The sublineage of PB2 exhibited a transient F/98-like sublineage between 2005 and 2009, after which the *PB2* genes of the viruses isolated post-2010 were found to belong mainly to the G1-like sublineage. The *M* gene belonged primarily to the BJ/94-like sublineage from 1999–2009; after 2010, the G1-like lineage *M* gene replaced the BJ/94-like sublineage. Studies have shown that H9N2 AIVs containing G1-like sublineage *PB2* and *M* genes have a competitive advantage in chickens, increasing virus infectivity and replication [57,58,59]. Therefore, these findings also explain the stable inheritance of the H9N2 AIV with the G1-like sublineage *PB2* and *M* genes in poultry.

A total of 117 genotypes (G1–G117) were identified on the basis of whole-genome phylogenetic analysis of H9N2 AIVs circulating in China from 1994–2003. Notably, genotype G57 appeared in 2007 and became the dominant genotype after 2010 [60]. Another study reported that 23 genotypes (A-W) were identified on the basis of the gene sublineage classification of H9N2 AIVs in China from 1996–2015 [28], with genotype S (generally equivalent to genotype G57) being the dominant genotype. In this study, the genotypes of H9N2 AIV in Guangxi from 1999–2023 were analysed using the classification method of Gu [28]. A total of 17 genotypes (G1–G17) were identified. Among them, 13 genotypes (G1–G13) were present in Guangxi before 2009. In 2010, G14 emerged in Guangxi; this genotype, also known as genotype S or G57 in China, was the dominant genotype in China after 2010. Since then, G14 has gradually replaced the other genotypes and has become the dominant genotype in poultry in Guangxi. Among the 17 genotypes of H9N2 AIV identified in Guangxi, 10 completely differed from those described by Gu [28], highlighting the diversity and complexity of genotypes in the evolutionary process of H9N2 AIV in Guangxi. The number of H9N2 AIV genotypes in poultry in China sharply increased from 2000–2005 [60]. Similarly, a variety of genotypes were present in Guangxi from 1999–2009. Two reasons for these increases were proposed by Li [60]. First, large-scale chicken farming facilitated the spread of various genotypes between chicken flocks and other hosts, such as quail and wild birds. Second, commercial inactivated vaccines were used to target viruses of A/chicken/Beijing/1/1994 and A/chicken/Shanghai/F/98 origin in 1998 in China, which accelerated gene reassortment of the virus to better adapt to poultry.

H9N2 AIV has a broad host range and has been reported to infect various mammals, including humans, swine, equines, canines and felines, in addition to poultry [61,62,63,64,65,66,67]. This study analysed H9N2 AIV infections across different hosts from 1999–2023 and reported cases of H9N2 AIV infection in mammals such as swine, equines and canines in Guangxi. The genotypes of H9N2 AIV from swine, equines and canines in Guangxi were consistent with those of H9N2 AIV from poultry during the same period, except for H9N2 AIV isolated from swine in 2007, which may be attributed to the infection of these mammals by avian H9N2 AIV, resulting in the same viral genotypes among these species. The H9N2 AIV strains isolated in this study from 2020–2023 were all obtained from LPMs. All these isolates belong to G14, which is the same genotype as the H9N2 AIV that has infected humans in China over the past 10 years. Recently, genotype S has been demonstrated to preferentially bind to α-2,6 sialic acid receptors [68]. Therefore, the potential public health significance of this genotype of H9N2 AIV in LPMs should be carefully studied.

This study provides a retrospective analysis of the genetic evolution of H9N2 AIV in Guangxi from 1999–2023, identifying 17 genotypes, with G14 being the dominant genotype in Guangxi since 2010. Owing to the limited number of H9N2 AIV isolates from neighbouring provinces and the absence of spatiotemporal dynamics analysis, this study did not determine whether the reassortments in Guangxi originated from local reassortment or were introduced from other regions. Guangxi has both intensive poultry farms and many live poultry markets, providing ideal conditions for coinfection and gene exchange among various H9N2 AIV genotypes. In addition, the location of Guangxi along the East Asian–Australasian migratory bird route allows migratory birds to introduce these viruses. Future studies should integrate the genes of H9N2 AIV from both poultry and wild birds in Guangxi and neighbouring provinces and use phylogeographic and migration models to determine the spatial and temporal origins and transmission pathways of reassortment events.

## Data Availability

The original contributions presented in this study are included in the article/Supplementary Material. Further inquiries can be directed to the corresponding authors.

## Supplementary Materials

The following supporting information can be downloaded at: https://www.mdpi.com/article/10.3390/microorganisms13112579/s1, Figure S1: ML trees of the *HA*, *NA*, *PB2*, *PB1*, *PA*, *NP*, *M*, and *NS* genes of H9N2 AIV; Figure S2: Genotypes of H9N2 AIVs in Guangxi from 1999–2023. Table S1: Information on H9N2 AIV strains isolated from live poultry markets in Guangxi from 2020–2023; Table S2: Detailed information of all reference H9N2 AIVs obtained from the GenBank database; Table S3: Antigenic site mutation frequency in H9N2 AIVs from Guangxi, 1999–2023; Table S4: Amino acid substitutions in the HA protein of H9N2 AIVs that contribute to antigenic drift; Table S5: Amino acid substitutions in H9N2 AIVs that contribute to enhanced pathogenicity, receptor binding, and transmissibility in mammals; Table S6: Amino acid at positions 234 and 235 (H9 numbering) of 86 H9N2 virus strains isolated in Guangxi during different periods; Table S7: Amino acid combinations at positions 234 and 235 (H9 numbering) of 86 H9N2 virus strains isolated in Guangxi from 1999 to 2023. Table S8: Antigenic site mutation frequency in NA protein of H9N2 AIV from Guangxi, 1999–2023.

## Abbreviations

The following abbreviations are used in this manuscript:

HA	Haemagglutinin
NA	Neuraminidase
PB2	Polymerase basic protein 2
PB1	Polymerase basic protein 1
PA	Polymerase acidic protein
NP	Nucleoprotein
M	Matrix protein
NS	Nonstructural protein
