# Supplementary material for: Genetic Evolution of H9N2 Avian Influenza Virus in Guangxi, China
Source: Microorganisms. 2025 Nov 12;13(11):2579. doi: 10.3390/microorganisms13112579 (PMC12654625; doi:10.3390/microorganisms13112579)
Supplement: Supplementary file 1 [file microorganisms-13-02579-s001.zip › Figure S1.pdf]

Figure S1

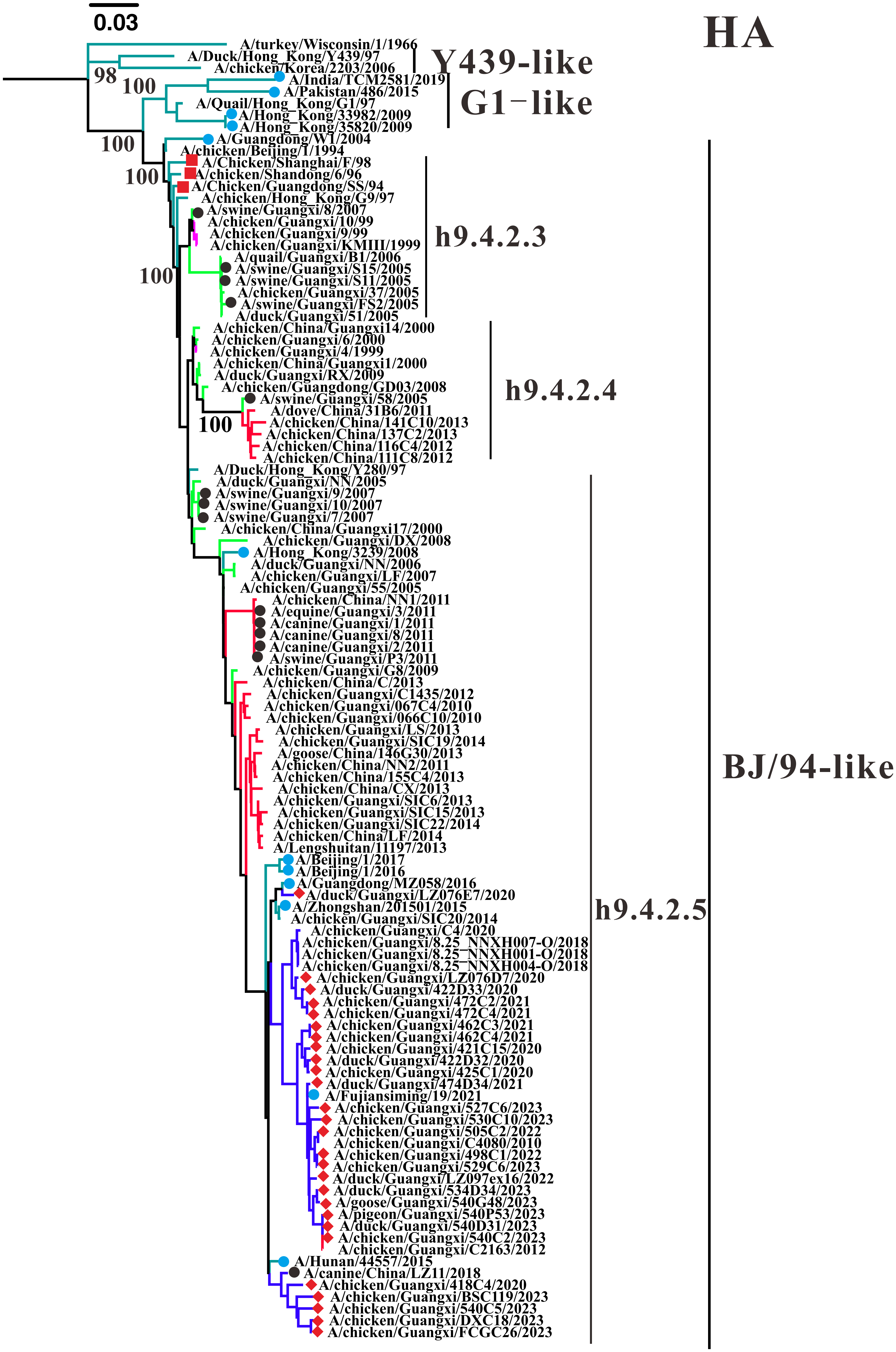
0.03
HA
A/turkey/Wisconsin/1/1966
A/Duck/Hong_Kong/Y439/97
A/chicken/Korea/2203/2006
Y439-like
98
100
A/India/TCM2581/2019
A/Pakistan/486/2015
A/Quail/Hong_Kong/G1/97
A/Hong_Kong/33982/2009
A/Hong_Kong/35820/2009
G1-like
100
A/Guangdong/W1/2004
A/chicken/Beijing/1/1994
A/Chicken/Shanghai/F/98
100
A/chicken/Shandong/6/96
A/Chicken/Guangdong/SS/94
A/chicken/Hong_Kong/G9/97
A/swine/Guangxi/8/2007
A/chicken/Guangxi/10/99
A/chicken/Guangxi/9/99
A/chicken/Guangxi/KMIII/1999
h9.4.2.3
A/quail/Guangxi/B1/2006
A/swine/Guangxi/S15/2005
100
A/swine/Guangxi/S11/2005
A/chicken/Guangxi/37/2005
A/swine/Guangxi/FS2/2005
A/duck/Guangxi/51/2005
A/chicken/China/Guangxi14/2000
A/chicken/Guangxi/6/2000
A/chicken/Guangxi/4/1999
A/chicken/China/Guangxi1/2000
A/duck/Guangxi/RX/2009
A/chicken/Guangdong/GD03/2008
h9.4.2.4
A/swine/Guangxi/58/2005
A/dove/China/31B6/2011
100
A/chicken/China/141C10/2013
A/chicken/China/137C2/2013
A/chicken/China/116C4/2012
A/chicken/China/111C8/2012
A/Duck/Hong_Kong/Y280/97
A/duck/Guangxi/NN/2005
A/swine/Guangxi/9/2007
A/swine/Guangxi/10/2007
A/swine/Guangxi/7/2007
A/chicken/China/Guangxi17/2000
A/chicken/Guangxi/DX/2008
A/Hong_Kong/3239/2008
A/duck/Guangxi/NN/2006
A/chicken/Guangxi/LF/2007
A/chicken/Guangxi/55/2005
A/chicken/China/NN1/2011
A/equine/Guangxi/3/2011
A/canine/Guangxi/1/2011
A/canine/Guangxi/8/2011
A/canine/Guangxi/2/2011
A/swine/Guangxi/P3/2011
A/chicken/Guangxi/G8/2009
A/chicken/China/C/2013
A/chicken/Guangxi/C1435/2012
A/chicken/Guangxi/067C4/2010
A/chicken/Guangxi/066C10/2010
A/chicken/Guangxi/LS/2013
A/chicken/Guangxi/SIC19/2014
A/goose/China/146G30/2013
A/chicken/China/NN2/2011
A/chicken/China/155C4/2013
A/chicken/China/CX/2013
A/chicken/Guangxi/SIC6/2013
A/chicken/Guangxi/SIC15/2013
A/chicken/Guangxi/SIC22/2014
A/chicken/China/LF/2014
A/Lengshuitan/11197/2013
BJ/94-like
A/Beijing/1/2017
A/Beijing/1/2016
A/Guangdong/MZ058/2016
A/duck/Guangxi/LZ076E7/2020
A/Zhongshan/201501/2015
A/chicken/Guangxi/SIC20/2014
h9.4.2.5
A/chicken/Guangxi/C4/2020
A/chicken/Guangxi/8.25_NNXH007-O/2018
A/chicken/Guangxi/8.25_NNXH001-O/2018
A/chicken/Guangxi/8.25_NNXH004-O/2018
A/chicken/Guangxi/LZ076D7/2020
A/duck/Guangxi/422D33/2020
A/chicken/Guangxi/472C2/2021
A/chicken/Guangxi/472C4/2021
A/chicken/Guangxi/462C3/2021
A/chicken/Guangxi/462C4/2021
A/chicken/Guangxi/421C15/2020
A/duck/Guangxi/422D32/2020
A/chicken/Guangxi/425C1/2020
A/duck/Guangxi/474D34/2021
A/Fujiansiming/19/2021
A/chicken/Guangxi/527C6/2023
A/chicken/Guangxi/530C10/2023
A/chicken/Guangxi/505C2/2022
A/chicken/Guangxi/C4080/2010
A/chicken/Guangxi/498C1/2022
A/chicken/Guangxi/529C6/2023
A/duck/Guangxi/LZ097ex16/2022
A/duck/Guangxi/534D34/2023
A/goose/Guangxi/540G48/2023
A/pigeon/Guangxi/540P53/2023
A/duck/Guangxi/540D31/2023
A/chicken/Guangxi/540C2/2023
A/chicken/Guangxi/C2163/2012
A/Hunan/44557/2015
A/canine/China/LZ11/2018
A/chicken/Guangxi/418C4/2020
A/chicken/Guangxi/BSC119/2023
A/chicken/Guangxi/540C5/2023
A/chicken/Guangxi/DXC18/2023
A/chicken/Guangxi/FCGC26/2023

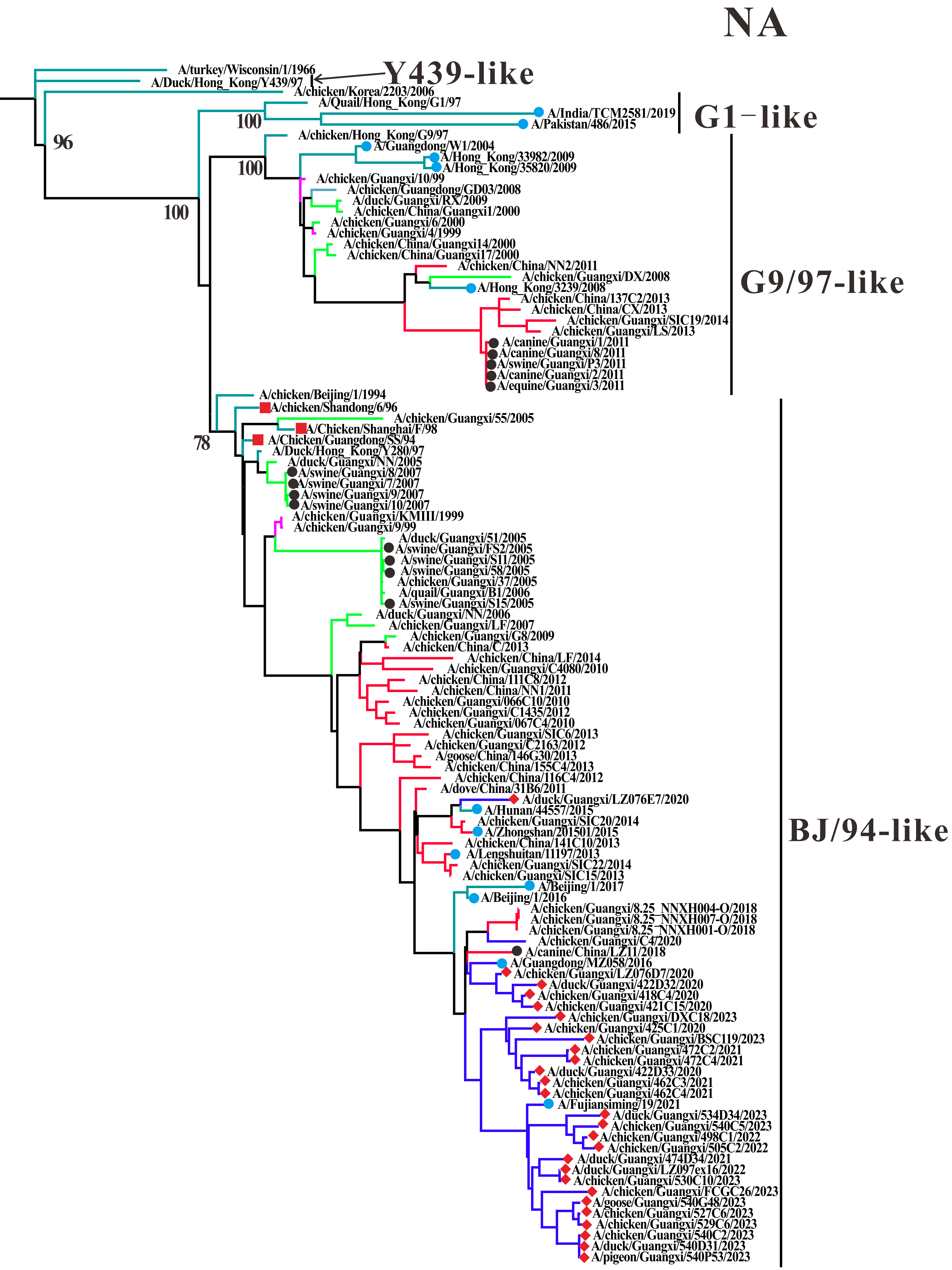
NA
Y439-like
G1-like
G9/97-like
BJ/94-like
A/turkey/Wisconsin/1/1966
A/Duck/Hong_Kong/Y439/97
A/chicken/Korea/2203/2006
A/Quail/Hong_Kong/G1/97
A/India/TCM2581/2019
A/Pakistan/486/2015
A/chicken/Hong_Kong/G9/97
A/Guangdong/W1/2004
A/Hong_Kong/33982/2009
A/Hong_Kong/35820/2009
A/chicken/Guangxi/10/99
A/chicken/Guangdong/GD03/2008
A/duck/Guangxi/RX/2009
A/chicken/China/Guangxi1/2000
A/chicken/Guangxi/6/2000
A/chicken/Guangxi/4/1999
A/chicken/China/Guangxi14/2000
A/chicken/China/Guangxi17/2000
A/chicken/China/NN2/2011
A/chicken/Guangxi/DX/2008
A/Hong_Kong/3239/2008
A/chicken/China/137C2/2013
A/chicken/China/CX/2013
A/chicken/Guangxi/SIC19/2014
A/chicken/Guangxi/LS/2013
A/canine/Guangxi/1/2011
A/canine/Guangxi/8/2011
A/swine/Guangxi/P3/2011
A/canine/Guangxi/2/2011
A/equine/Guangxi/3/2011
A/chicken/Beijing/1/1994
A/chicken/Shandong/6/96
A/chicken/Guangxi/55/2005
A/Chicken/Shanghai/F/98
A/Chicken/Guangdong/SS/94
A/Duck/Hong_Kong/Y280/97
A/duck/Guangxi/NN/2005
A/swine/Guangxi/8/2007
A/swine/Guangxi/7/2007
A/swine/Guangxi/9/2007
A/swine/Guangxi/10/2007
A/chicken/Guangxi/KMIII/1999
A/chicken/Guangxi/9/99
A/duck/Guangxi/51/2005
A/swine/Guangxi/FS2/2005
A/swine/Guangxi/S11/2005
A/swine/Guangxi/58/2005
A/chicken/Guangxi/37/2005
A/quail/Guangxi/B1/2006
A/swine/Guangxi/S15/2005
A/duck/Guangxi/NN/2006
A/chicken/Guangxi/LF/2007
A/chicken/Guangxi/G8/2009
A/chicken/China/C/2013
A/chicken/China/LF/2014
A/chicken/Guangxi/C4080/2010
A/chicken/China/111C8/2012
A/chicken/China/NN1/2011
A/chicken/Guangxi/066C10/2010
A/chicken/Guangxi/C1435/2012
A/chicken/Guangxi/067C4/2010
A/chicken/Guangxi/SIC6/2013
A/chicken/Guangxi/C2163/2012
A/goose/China/146G30/2013
A/chicken/China/155C4/2013
A/chicken/China/116C4/2012
A/dove/China/31B6/2011
A/duck/Guangxi/LZ076E7/2020
A/Hunan/44557/2015
A/chicken/Guangxi/SIC20/2014
A/Zhongshan/201501/2015
A/chicken/China/141C10/2013
A/Lengshuitan/11197/2013
A/chicken/Guangxi/SIC22/2014
A/chicken/Guangxi/SIC15/2013
A/Beijing/1/2017
A/Beijing/1/2016
A/chicken/Guangxi/8.25_NNXH004-O/2018
A/chicken/Guangxi/8.25_NNXH007-O/2018
A/chicken/Guangxi/8.25_NNXH001-O/2018
A/chicken/Guangxi/C4/2020
A/canine/China/LZ11/2018
A/Guangdong/MZ058/2016
A/chicken/Guangxi/LZ076D7/2020
A/duck/Guangxi/422D32/2020
A/chicken/Guangxi/418C4/2020
A/chicken/Guangxi/421C15/2020
A/chicken/Guangxi/DXC18/2023
A/chicken/Guangxi/425C1/2020
A/chicken/Guangxi/BSC119/2023
A/chicken/Guangxi/472C2/2021
A/chicken/Guangxi/472C4/2021
A/duck/Guangxi/422D33/2020
A/chicken/Guangxi/462C3/2021
A/chicken/Guangxi/462C4/2021
A/Fujiansiming/19/2021
A/duck/Guangxi/534D34/2023
A/chicken/Guangxi/540C5/2023
A/chicken/Guangxi/498C1/2022
A/chicken/Guangxi/505C2/2022
A/duck/Guangxi/474D34/2021
A/duck/Guangxi/LZ097ex16/2022
A/chicken/Guangxi/530C10/2023
A/chicken/Guangxi/FCGC26/2023
A/goose/Guangxi/540G48/2023
A/chicken/Guangxi/527C6/2023
A/chicken/Guangxi/529C6/2023
A/chicken/Guangxi/540C2/2023
A/duck/Guangxi/540D31/2023
A/pigeon/Guangxi/540P53/2023
96
100
100
100
78

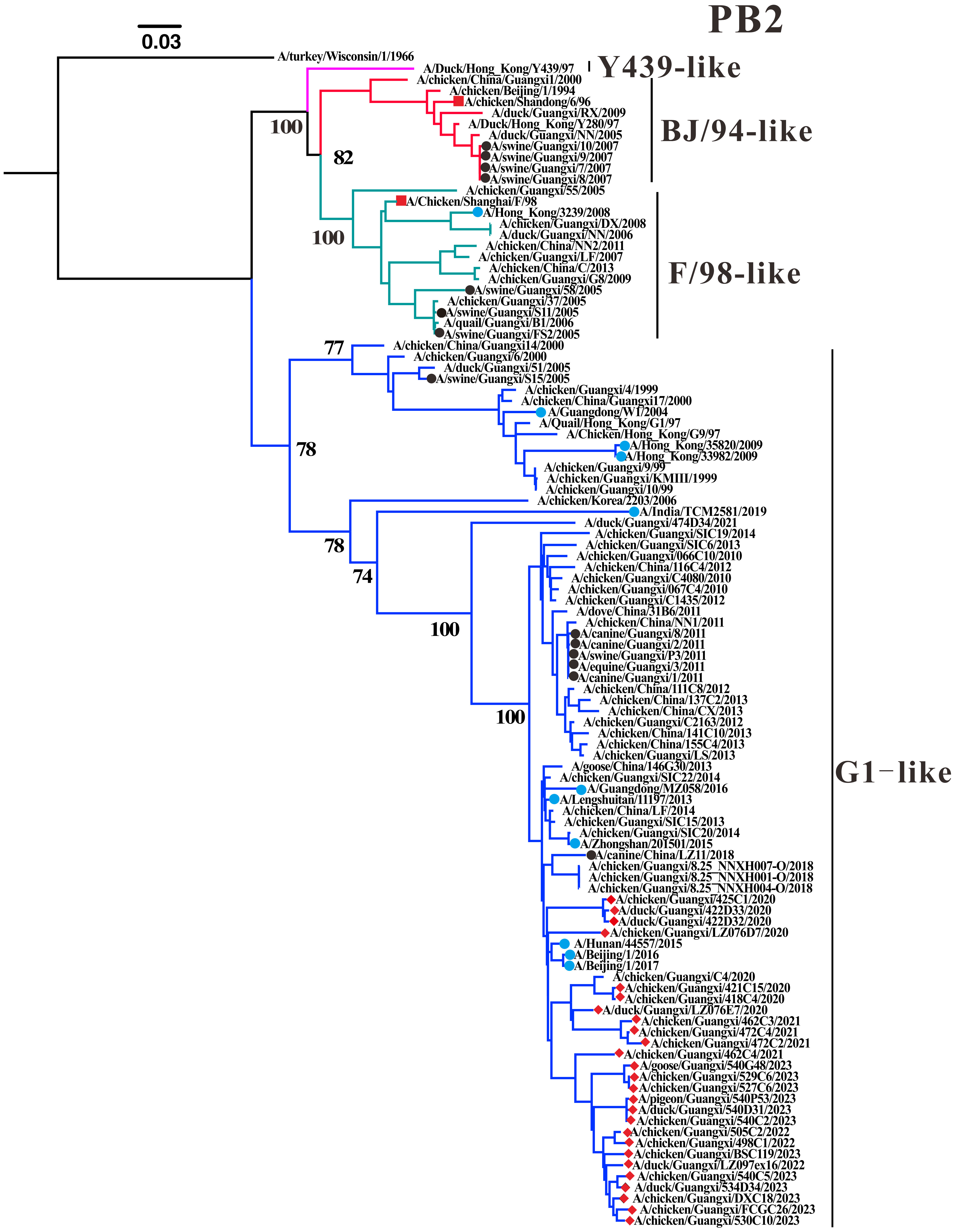

PB2
0.03
A/turkey/Wisconsin/1/1966
A/Duck/Hong_Kong/Y439/97
Y439-like
A/chicken/China/Guangxi1/2000
A/chicken/Beijing/1/1994
A/chicken/Shandong/6/96
A/duck/Guangxi/RX/2009
A/Duck/Hong_Kong/Y280/97
A/duck/Guangxi/NN/2005
A/swine/Guangxi/10/2007
A/swine/Guangxi/9/2007
A/swine/Guangxi/7/2007
A/swine/Guangxi/8/2007
BJ/94-like
100
82
A/chicken/Guangxi/55/2005
A/Chicken/Shanghai/F/98
A/Hong_Kong/3239/2008
A/chicken/Guangxi/DX/2008
A/duck/Guangxi/NN/2006
A/chicken/China/NN2/2011
A/chicken/Guangxi/LF/2007
A/chicken/China/C/2013
A/chicken/Guangxi/G8/2009
A/swine/Guangxi/58/2005
A/chicken/Guangxi/37/2005
A/swine/Guangxi/S11/2005
A/quail/Guangxi/B1/2006
A/swine/Guangxi/FS2/2005
100
F/98-like
77
A/chicken/China/Guangxi14/2000
A/chicken/Guangxi/6/2000
A/duck/Guangxi/51/2005
A/swine/Guangxi/S15/2005
A/chicken/Guangxi/4/1999
A/chicken/China/Guangxi17/2000
A/Guangdong/W1/2004
A/Quail/Hong_Kong/G1/97
A/Chicken/Hong_Kong/G9/97
A/Hong_Kong/35820/2009
A/Hong_Kong/33982/2009
A/chicken/Guangxi/9/99
A/chicken/Guangxi/KMIII/1999
A/chicken/Guangxi/10/99
78
A/chicken/Korea/2203/2006
A/India/TCM2581/2019
A/duck/Guangxi/474D34/2021
A/chicken/Guangxi/SIC19/2014
A/chicken/Guangxi/SIC6/2013
A/chicken/Guangxi/066C10/2010
A/chicken/China/116C4/2012
A/chicken/Guangxi/C4080/2010
A/chicken/Guangxi/067C4/2010
A/chicken/Guangxi/C1435/2012
A/dove/China/31B6/2011
A/chicken/China/NN1/2011
A/canine/Guangxi/8/2011
A/canine/Guangxi/2/2011
A/swine/Guangxi/P3/2011
A/equine/Guangxi/3/2011
A/canine/Guangxi/1/2011
A/chicken/China/111C8/2012
A/chicken/China/137C2/2013
A/chicken/China/CX/2013
A/chicken/Guangxi/C2163/2012
A/chicken/China/141C10/2013
A/chicken/China/155C4/2013
A/chicken/Guangxi/LS/2013
78
74
100
100
A/goose/China/146G30/2013
A/chicken/Guangxi/SIC22/2014
A/Guangdong/MZ058/2016
A/Lengshuitan/11197/2013
A/chicken/China/LF/2014
A/chicken/Guangxi/SIC15/2013
A/chicken/Guangxi/SIC20/2014
A/Zhongshan/201501/2015
A/canine/China/LZ11/2018
A/chicken/Guangxi/8.25_NNXH007-O/2018
A/chicken/Guangxi/8.25_NNXH001-O/2018
A/chicken/Guangxi/8.25_NNXH004-O/2018
A/chicken/Guangxi/425C1/2020
A/duck/Guangxi/422D33/2020
A/duck/Guangxi/422D32/2020
A/chicken/Guangxi/LZ076D7/2020
A/Hunan/44557/2015
A/Beijing/1/2016
A/Beijing/1/2017
A/chicken/Guangxi/C4/2020
A/chicken/Guangxi/421C15/2020
A/chicken/Guangxi/418C4/2020
A/duck/Guangxi/LZ076E7/2020
A/chicken/Guangxi/462C3/2021
A/chicken/Guangxi/472C4/2021
A/chicken/Guangxi/472C2/2021
A/chicken/Guangxi/462C4/2021
A/goose/Guangxi/540G48/2023
A/chicken/Guangxi/529C6/2023
A/chicken/Guangxi/527C6/2023
A/pigeon/Guangxi/540P53/2023
A/duck/Guangxi/540D31/2023
A/chicken/Guangxi/540C2/2023
A/chicken/Guangxi/505C2/2022
A/chicken/Guangxi/498C1/2022
A/chicken/Guangxi/BSC119/2023
A/duck/Guangxi/LZ097ex16/2022
A/chicken/Guangxi/540C5/2023
A/duck/Guangxi/534D34/2023
A/chicken/Guangxi/DXC18/2023
A/chicken/Guangxi/FCGC26/2023
A/chicken/Guangxi/530C10/2023
G1-like

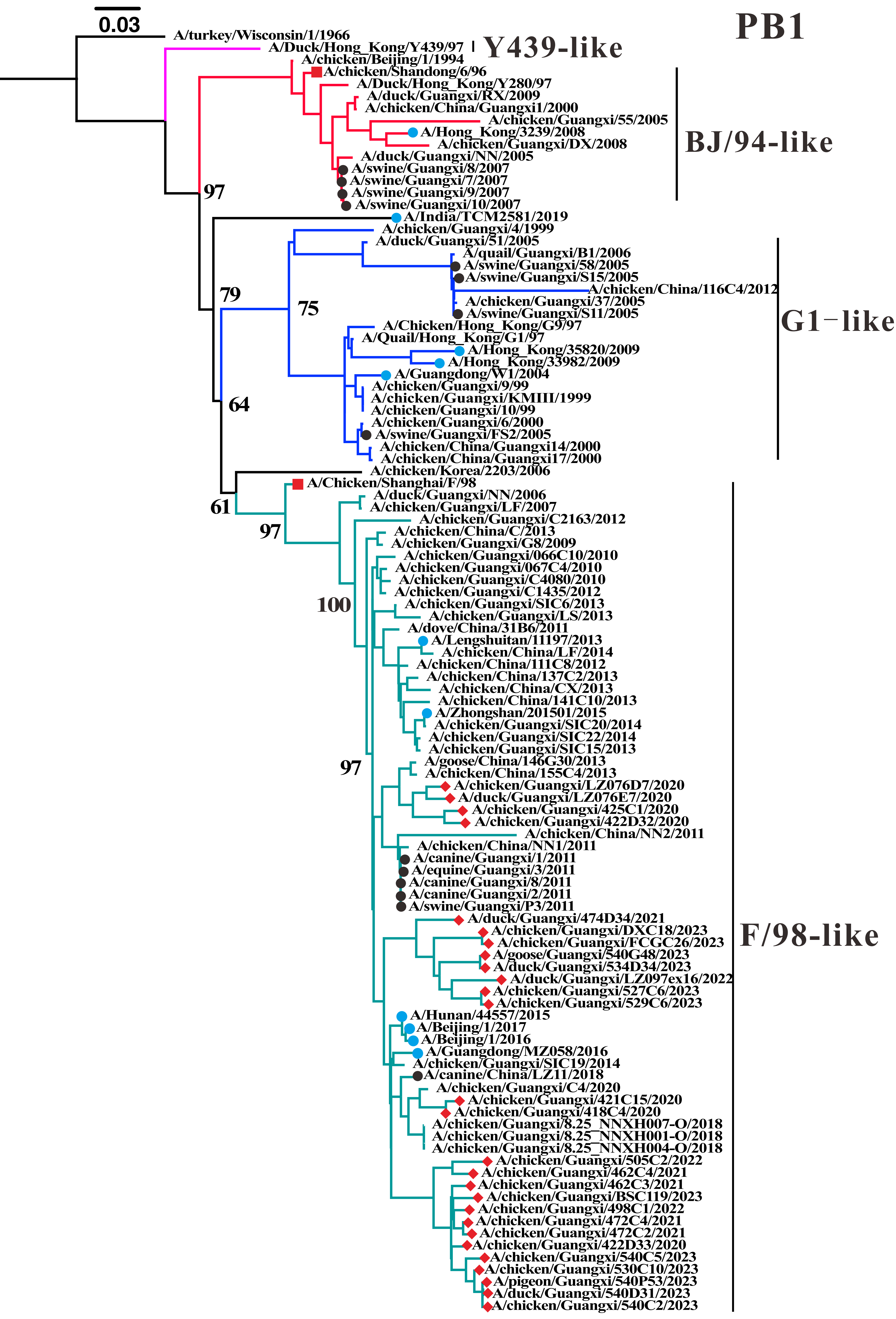

PB1
0.03
A/turkey/Wisconsin/1/1966
A/Duck/Hong_Kong/Y439/97
Y439-like
A/chicken/Beijing/1/1994
A/chicken/Shandong/6/96
A/Duck/Hong_Kong/Y280/97
A/duck/Guangxi/RX/2009
A/chicken/China/Guangxi1/2000
A/chicken/Guangxi/55/2005
A/Hong_Kong/3239/2008
A/chicken/Guangxi/DX/2008
BJ/94-like
A/duck/Guangxi/NN/2005
A/swine/Guangxi/8/2007
A/swine/Guangxi/7/2007
A/swine/Guangxi/9/2007
A/swine/Guangxi/10/2007
97
A/India/TCM2581/2019
A/chicken/Guangxi/4/1999
A/duck/Guangxi/51/2005
A/quail/Guangxi/B1/2006
A/swine/Guangxi/58/2005
A/swine/Guangxi/S15/2005
A/chicken/China/116C4/2012
79
A/chicken/Guangxi/37/2005
75
A/swine/Guangxi/S11/2005
G1-like
A/Chicken/Hong_Kong/G9/97
A/Quail/Hong_Kong/G1/97
A/Hong_Kong/35820/2009
A/Hong_Kong/33982/2009
A/Guangdong/W1/2004
A/chicken/Guangxi/9/99
A/chicken/Guangxi/KMIII/1999
64
A/chicken/Guangxi/10/99
A/chicken/Guangxi/6/2000
A/swine/Guangxi/FS2/2005
A/chicken/China/Guangxi14/2000
A/chicken/China/Guangxi17/2000
A/chicken/Korea/2203/2006
A/Chicken/Shanghai/F/98
61
A/duck/Guangxi/NN/2006
A/chicken/Guangxi/LF/2007
97
A/chicken/Guangxi/C2163/2012
A/chicken/China/C/2013
A/chicken/Guangxi/G8/2009
A/chicken/Guangxi/066C10/2010
A/chicken/Guangxi/067C4/2010
A/chicken/Guangxi/C4080/2010
A/chicken/Guangxi/C1435/2012
100
A/chicken/Guangxi/SIC6/2013
A/chicken/Guangxi/LS/2013
A/dove/China/31B6/2011
A/Lengshuitan/11197/2013
A/chicken/China/LF/2014
A/chicken/China/111C8/2012
A/chicken/China/137C2/2013
A/chicken/China/CX/2013
A/chicken/China/141C10/2013
A/Zhongshan/201501/2015
A/chicken/Guangxi/SIC20/2014
A/chicken/Guangxi/SIC22/2014
A/chicken/Guangxi/SIC15/2013
A/goose/China/146G30/2013
97
A/chicken/China/155C4/2013
A/chicken/Guangxi/LZ076D7/2020
A/duck/Guangxi/LZ076E7/2020
A/chicken/Guangxi/425C1/2020
A/chicken/Guangxi/422D32/2020
A/chicken/China/NN2/2011
A/chicken/China/NN1/2011
A/canine/Guangxi/1/2011
A/equine/Guangxi/3/2011
A/canine/Guangxi/8/2011
A/canine/Guangxi/2/2011
A/swine/Guangxi/P3/2011
A/duck/Guangxi/474D34/2021
A/chicken/Guangxi/DXC18/2023
A/chicken/Guangxi/FCGC26/2023
F/98-like
A/goose/Guangxi/540G48/2023
A/duck/Guangxi/534D34/2023
A/duck/Guangxi/LZ097ex16/2022
A/chicken/Guangxi/527C6/2023
A/chicken/Guangxi/529C6/2023
A/Hunan/44557/2015
A/Beijing/1/2017
A/Beijing/1/2016
A/Guangdong/MZ058/2016
A/chicken/Guangxi/SIC19/2014
A/canine/China/LZ11/2018
A/chicken/Guangxi/C4/2020
A/chicken/Guangxi/421C15/2020
A/chicken/Guangxi/418C4/2020
A/chicken/Guangxi/8.25_NNXH007-O/2018
A/chicken/Guangxi/8.25_NNXH001-O/2018
A/chicken/Guangxi/8.25_NNXH004-O/2018
A/chicken/Guangxi/505C2/2022
A/chicken/Guangxi/462C4/2021
A/chicken/Guangxi/462C3/2021
A/chicken/Guangxi/BSC119/2023
A/chicken/Guangxi/498C1/2022
A/chicken/Guangxi/472C4/2021
A/chicken/Guangxi/472C2/2021
A/chicken/Guangxi/422D33/2020
A/chicken/Guangxi/540C5/2023
A/chicken/Guangxi/530C10/2023
A/pigeon/Guangxi/540P53/2023
A/duck/Guangxi/540D31/2023
A/chicken/Guangxi/540C2/2023

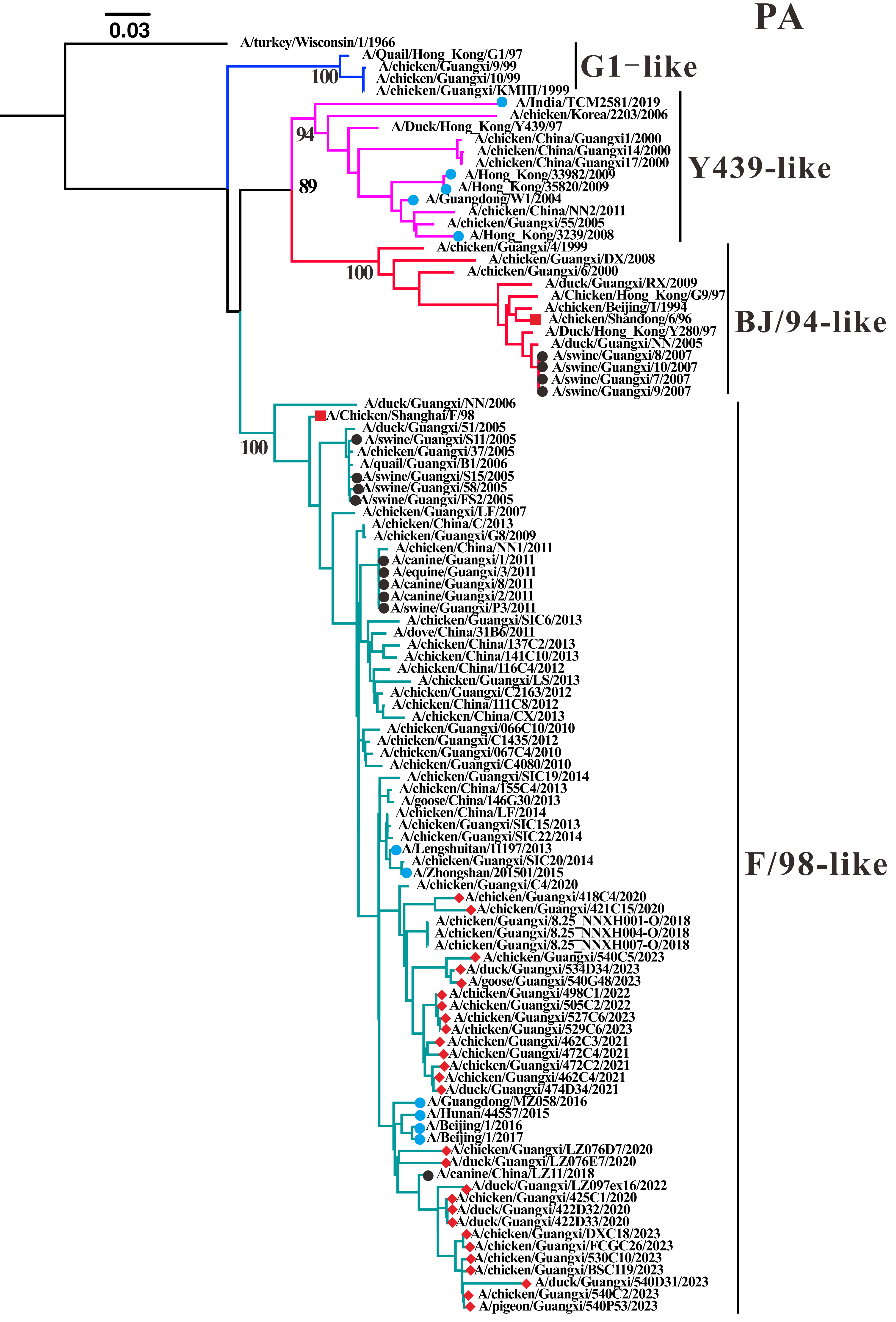
PA
0.03
A/turkey/Wisconsin/1/1966
A/Quail/Hong_Kong/G1/97
A/chicken/Guangxi/9/99
A/chicken/Guangxi/10/99
A/chicken/Guangxi/KMIII/1999
100
G1-like
A/India/TCM2581/2019
A/chicken/Korea/2203/2006
94
A/Duck/Hong_Kong/Y439/97
A/chicken/China/Guangxi1/2000
A/chicken/China/Guangxi14/2000
A/chicken/China/Guangxi17/2000
A/Hong_Kong/33982/2009
89
A/Hong_Kong/35820/2009
A/Guangdong/W1/2004
A/chicken/China/NN2/2011
A/chicken/Guangxi/55/2005
A/Hong_Kong/3239/2008
Y439-like
A/chicken/Guangxi/4/1999
A/chicken/Guangxi/DX/2008
100
A/chicken/Guangxi/6/2000
A/duck/Guangxi/RX/2009
A/Chicken/Hong_Kong/G9/97
A/chicken/Beijing/1/1994
A/chicken/Shandong/6/96
A/Duck/Hong_Kong/Y280/97
A/duck/Guangxi/NN/2005
A/swine/Guangxi/8/2007
A/swine/Guangxi/10/2007
A/swine/Guangxi/7/2007
A/swine/Guangxi/9/2007
BJ/94-like
A/duck/Guangxi/NN/2006
A/Chicken/Shanghai/F/98
A/duck/Guangxi/51/2005
100
A/swine/Guangxi/S11/2005
A/chicken/Guangxi/37/2005
A/quail/Guangxi/B1/2006
A/swine/Guangxi/S15/2005
A/swine/Guangxi/58/2005
A/swine/Guangxi/FS2/2005
A/chicken/Guangxi/LF/2007
A/chicken/China/C/2013
A/chicken/Guangxi/G8/2009
A/chicken/China/NN1/2011
A/canine/Guangxi/1/2011
A/equine/Guangxi/3/2011
A/canine/Guangxi/8/2011
A/canine/Guangxi/2/2011
A/swine/Guangxi/P3/2011
A/chicken/Guangxi/SIC6/2013
A/dove/China/31B6/2011
A/chicken/China/137C2/2013
A/chicken/China/141C10/2013
A/chicken/China/116C4/2012
A/chicken/Guangxi/LS/2013
A/chicken/Guangxi/C2163/2012
A/chicken/China/111C8/2012
A/chicken/China/CX/2013
A/chicken/Guangxi/066C10/2010
A/chicken/Guangxi/C1435/2012
A/chicken/Guangxi/067C4/2010
A/chicken/Guangxi/C4080/2010
A/chicken/Guangxi/SIC19/2014
A/chicken/China/155C4/2013
A/goose/China/146G30/2013
A/chicken/China/LF/2014
A/chicken/Guangxi/SIC15/2013
A/chicken/Guangxi/SIC22/2014
A/Lengshuitan/11197/2013
A/chicken/Guangxi/SIC20/2014
A/Zhongshan/201501/2015
A/chicken/Guangxi/C4/2020
A/chicken/Guangxi/418C4/2020
A/chicken/Guangxi/421C15/2020
A/chicken/Guangxi/8.25_NNXH001-O/2018
A/chicken/Guangxi/8.25_NNXH004-O/2018
A/chicken/Guangxi/8.25_NNXH007-O/2018
A/chicken/Guangxi/540C5/2023
A/duck/Guangxi/534D34/2023
A/goose/Guangxi/540G48/2023
A/chicken/Guangxi/498C1/2022
A/chicken/Guangxi/505C2/2022
A/chicken/Guangxi/527C6/2023
A/chicken/Guangxi/529C6/2023
A/chicken/Guangxi/462C3/2021
A/chicken/Guangxi/472C4/2021
A/chicken/Guangxi/472C2/2021
A/chicken/Guangxi/462C4/2021
A/duck/Guangxi/474D34/2021
A/Guangdong/MZ058/2016
A/Hunan/44557/2015
A/Beijing/1/2016
A/Beijing/1/2017
A/chicken/Guangxi/LZ076D7/2020
A/duck/Guangxi/LZ076E7/2020
A/canine/China/LZ11/2018
A/duck/Guangxi/LZ097ex16/2022
A/chicken/Guangxi/425C1/2020
A/duck/Guangxi/422D32/2020
A/duck/Guangxi/422D33/2020
A/chicken/Guangxi/DXC18/2023
A/chicken/Guangxi/FCGC26/2023
A/chicken/Guangxi/530C10/2023
A/chicken/Guangxi/BSC119/2023
A/duck/Guangxi/540D31/2023
A/chicken/Guangxi/540C2/2023
A/pigeon/Guangxi/540P53/2023
F/98-like

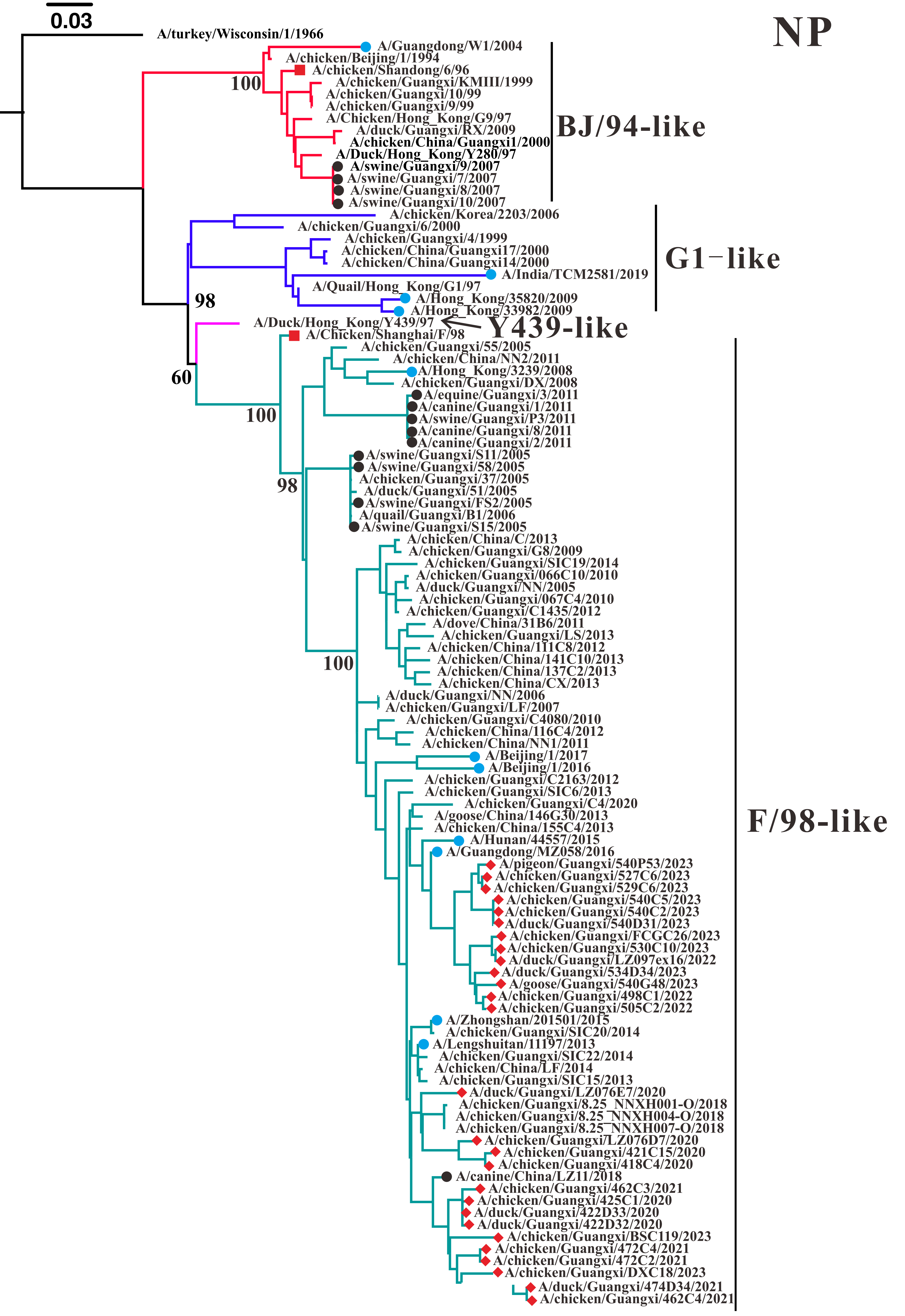

0.03
NP
A/turkey/Wisconsin/1/1966
A/Guangdong/W1/2004
A/chicken/Beijing/1/1994
A/chicken/Shandong/6/96
A/chicken/Guangxi/KMIII/1999
A/chicken/Guangxi/10/99
A/chicken/Guangxi/9/99
A/Chicken/Hong_Kong/G9/97
A/duck/Guangxi/RX/2009
A/chicken/China/Guangxi1/2000
A/Duck/Hong_Kong/Y280/97
A/swine/Guangxi/9/2007
A/swine/Guangxi/7/2007
A/swine/Guangxi/8/2007
A/swine/Guangxi/10/2007
100
BJ/94-like
A/chicken/Korea/2203/2006
A/chicken/Guangxi/6/2000
A/chicken/Guangxi/4/1999
A/chicken/China/Guangxi17/2000
A/chicken/China/Guangxi14/2000
A/India/TCM2581/2019
A/Quail/Hong_Kong/G1/97
A/Hong_Kong/35820/2009
A/Hong_Kong/33982/2009
98
G1-like
A/Duck/Hong_Kong/Y439/97
Y439-like
A/Chicken/Shanghai/F/98
60
100
A/chicken/Guangxi/55/2005
A/chicken/China/NN2/2011
A/Hong_Kong/3239/2008
A/chicken/Guangxi/DX/2008
A/equine/Guangxi/3/2011
A/canine/Guangxi/1/2011
A/swine/Guangxi/P3/2011
A/canine/Guangxi/8/2011
A/canine/Guangxi/2/2011
A/swine/Guangxi/S11/2005
A/swine/Guangxi/58/2005
A/chicken/Guangxi/37/2005
A/duck/Guangxi/51/2005
A/swine/Guangxi/FS2/2005
A/quail/Guangxi/B1/2006
A/swine/Guangxi/S15/2005
98
A/chicken/China/C/2013
A/chicken/Guangxi/G8/2009
A/chicken/Guangxi/SIC19/2014
A/chicken/Guangxi/066C10/2010
A/duck/Guangxi/NN/2005
A/chicken/Guangxi/067C4/2010
A/chicken/Guangxi/C1435/2012
A/dove/China/31B6/2011
A/chicken/Guangxi/LS/2013
A/chicken/China/111C8/2012
A/chicken/China/141C10/2013
A/chicken/China/137C2/2013
A/chicken/China/CX/2013
100
A/duck/Guangxi/NN/2006
A/chicken/Guangxi/LF/2007
A/chicken/Guangxi/C4080/2010
A/chicken/China/116C4/2012
A/chicken/China/NN1/2011
A/Beijing/1/2017
A/Beijing/1/2016
A/chicken/Guangxi/C2163/2012
A/chicken/Guangxi/SIC6/2013
A/chicken/Guangxi/C4/2020
A/goose/China/146G30/2013
A/chicken/China/155C4/2013
A/Hunan/44557/2015
A/Guangdong/MZ058/2016
A/pigeon/Guangxi/540P53/2023
A/chicken/Guangxi/527C6/2023
A/chicken/Guangxi/529C6/2023
A/chicken/Guangxi/540C5/2023
A/chicken/Guangxi/540C2/2023
A/duck/Guangxi/540D31/2023
A/chicken/Guangxi/FCGC26/2023
A/chicken/Guangxi/530C10/2023
A/duck/Guangxi/LZ097ex16/2022
A/duck/Guangxi/534D34/2023
A/goose/Guangxi/540G48/2023
A/chicken/Guangxi/498C1/2022
A/chicken/Guangxi/505C2/2022
A/Zhongshan/201501/2015
A/chicken/Guangxi/SIC20/2014
A/Lengshuitan/11197/2013
A/chicken/Guangxi/SIC22/2014
A/chicken/China/LF/2014
A/chicken/Guangxi/SIC15/2013
A/duck/Guangxi/LZ076E7/2020
A/chicken/Guangxi/8.25_NNXH001-O/2018
A/chicken/Guangxi/8.25_NNXH004-O/2018
A/chicken/Guangxi/8.25_NNXH007-O/2018
A/chicken/Guangxi/LZ076D7/2020
A/chicken/Guangxi/421C15/2020
A/chicken/Guangxi/418C4/2020
A/canine/China/LZ11/2018
A/chicken/Guangxi/462C3/2021
A/chicken/Guangxi/425C1/2020
A/duck/Guangxi/422D33/2020
A/duck/Guangxi/422D32/2020
A/chicken/Guangxi/BSC119/2023
A/chicken/Guangxi/472C4/2021
A/chicken/Guangxi/472C2/2021
A/chicken/Guangxi/DXC18/2023
A/duck/Guangxi/474D34/2021
A/chicken/Guangxi/462C4/2021
F/98-like

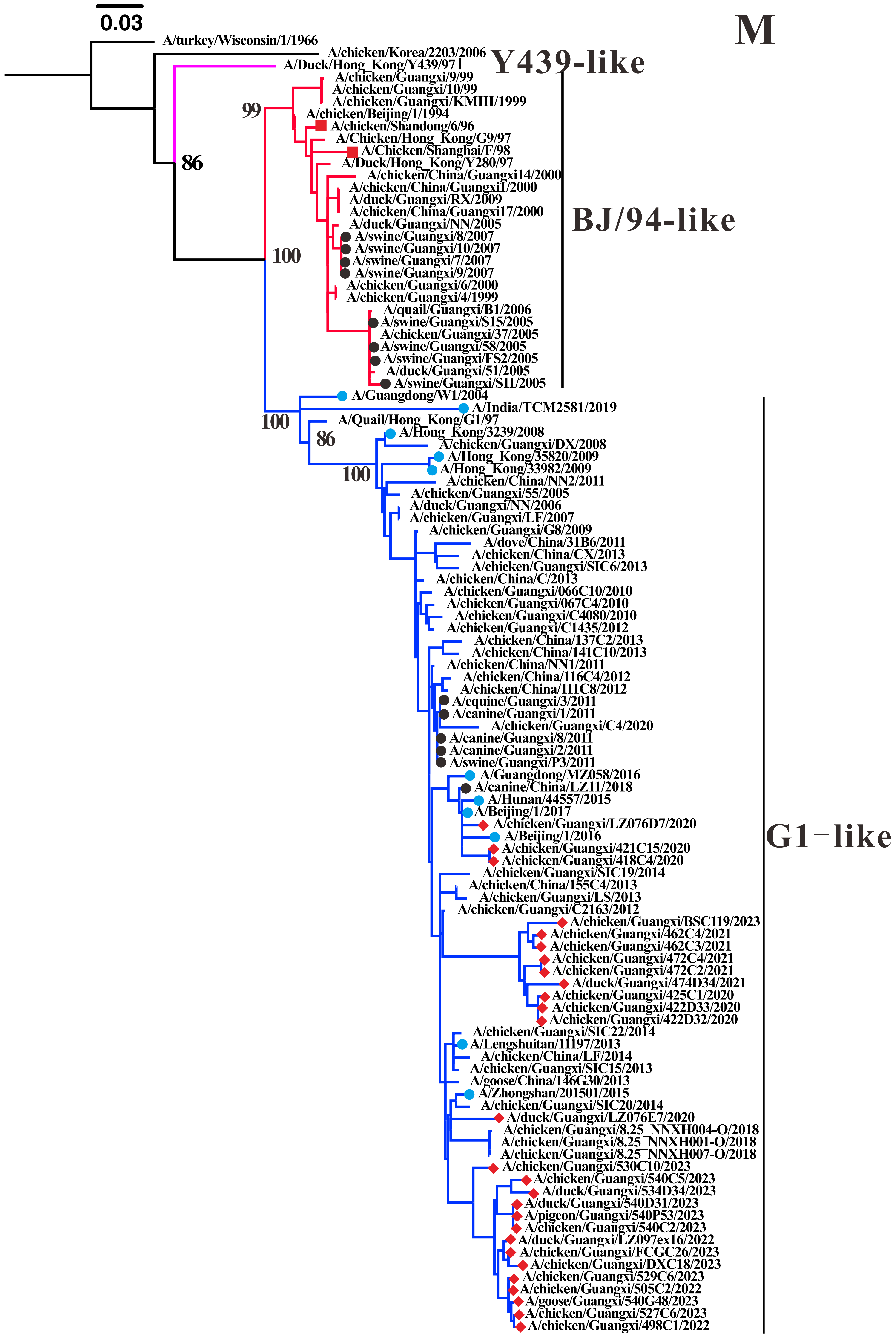

M
0.03
A/turkey/Wisconsin/1/1966
A/chicken/Korea/2203/2006
A/Duck/Hong_Kong/Y439/97
Y439-like
A/chicken/Guangxi/9/99
A/chicken/Guangxi/10/99
A/chicken/Guangxi/KMIII/1999
99
A/chicken/Beijing/1/1994
A/chicken/Shandong/6/96
A/Chicken/Hong_Kong/G9/97
A/Chicken/Shanghai/F/98
86
A/Duck/Hong_Kong/Y280/97
A/chicken/China/Guangxi14/2000
A/chicken/China/Guangxi1/2000
A/duck/Guangxi/RX/2009
A/chicken/China/Guangxi17/2000
BJ/94-like
A/duck/Guangxi/NN/2005
A/swine/Guangxi/8/2007
A/swine/Guangxi/10/2007
100
A/swine/Guangxi/7/2007
A/swine/Guangxi/9/2007
A/chicken/Guangxi/6/2000
A/chicken/Guangxi/4/1999
A/quail/Guangxi/B1/2006
A/swine/Guangxi/S15/2005
A/chicken/Guangxi/37/2005
A/swine/Guangxi/58/2005
A/swine/Guangxi/FS2/2005
A/duck/Guangxi/51/2005
A/swine/Guangxi/S11/2005
A/Guangdong/W1/2004
A/India/TCM2581/2019
100
A/Quail/Hong_Kong/G1/97
86
A/Hong_Kong/3239/2008
A/chicken/Guangxi/DX/2008
A/Hong_Kong/35820/2009
A/Hong_Kong/33982/2009
100
A/chicken/China/NN2/2011
A/chicken/Guangxi/55/2005
A/duck/Guangxi/NN/2006
A/chicken/Guangxi/LF/2007
A/chicken/Guangxi/G8/2009
A/dove/China/31B6/2011
A/chicken/China/CX/2013
A/chicken/Guangxi/SIC6/2013
A/chicken/China/C/2013
A/chicken/Guangxi/066C10/2010
A/chicken/Guangxi/067C4/2010
A/chicken/Guangxi/C4080/2010
A/chicken/Guangxi/C1435/2012
A/chicken/China/137C2/2013
A/chicken/China/141C10/2013
A/chicken/China/NN1/2011
A/chicken/China/116C4/2012
A/chicken/China/111C8/2012
A/equine/Guangxi/3/2011
A/canine/Guangxi/1/2011
A/chicken/Guangxi/C4/2020
A/canine/Guangxi/8/2011
A/canine/Guangxi/2/2011
A/swine/Guangxi/P3/2011
A/Guangdong/MZ058/2016
A/canine/China/LZ11/2018
A/Hunan/44557/2015
A/Beijing/1/2017
A/chicken/Guangxi/LZ076D7/2020
A/Beijing/1/2016
G1-like
A/chicken/Guangxi/421C15/2020
A/chicken/Guangxi/418C4/2020
A/chicken/Guangxi/SIC19/2014
A/chicken/China/155C4/2013
A/chicken/Guangxi/LS/2013
A/chicken/Guangxi/C2163/2012
A/chicken/Guangxi/BSC119/2023
A/chicken/Guangxi/462C4/2021
A/chicken/Guangxi/462C3/2021
A/chicken/Guangxi/472C4/2021
A/chicken/Guangxi/472C2/2021
A/duck/Guangxi/474D34/2021
A/chicken/Guangxi/425C1/2020
A/chicken/Guangxi/422D33/2020
A/chicken/Guangxi/422D32/2020
A/chicken/Guangxi/SIC22/2014
A/Lengshuitan/11197/2013
A/chicken/China/LF/2014
A/chicken/Guangxi/SIC15/2013
A/goose/China/146G30/2013
A/Zhongshan/201501/2015
A/chicken/Guangxi/SIC20/2014
A/duck/Guangxi/LZ076E7/2020
A/chicken/Guangxi/8.25_NNXH004-O/2018
A/chicken/Guangxi/8.25_NNXH001-O/2018
A/chicken/Guangxi/8.25_NNXH007-O/2018
A/chicken/Guangxi/530C10/2023
A/chicken/Guangxi/540C5/2023
A/duck/Guangxi/534D34/2023
A/duck/Guangxi/540D31/2023
A/pigeon/Guangxi/540P53/2023
A/chicken/Guangxi/540C2/2023
A/duck/Guangxi/LZ097ex16/2022
A/chicken/Guangxi/FCGC26/2023
A/chicken/Guangxi/DXC18/2023
A/chicken/Guangxi/529C6/2023
A/chicken/Guangxi/505C2/2022
A/goose/Guangxi/540G48/2023
A/chicken/Guangxi/527C6/2023
A/chicken/Guangxi/498C1/2022

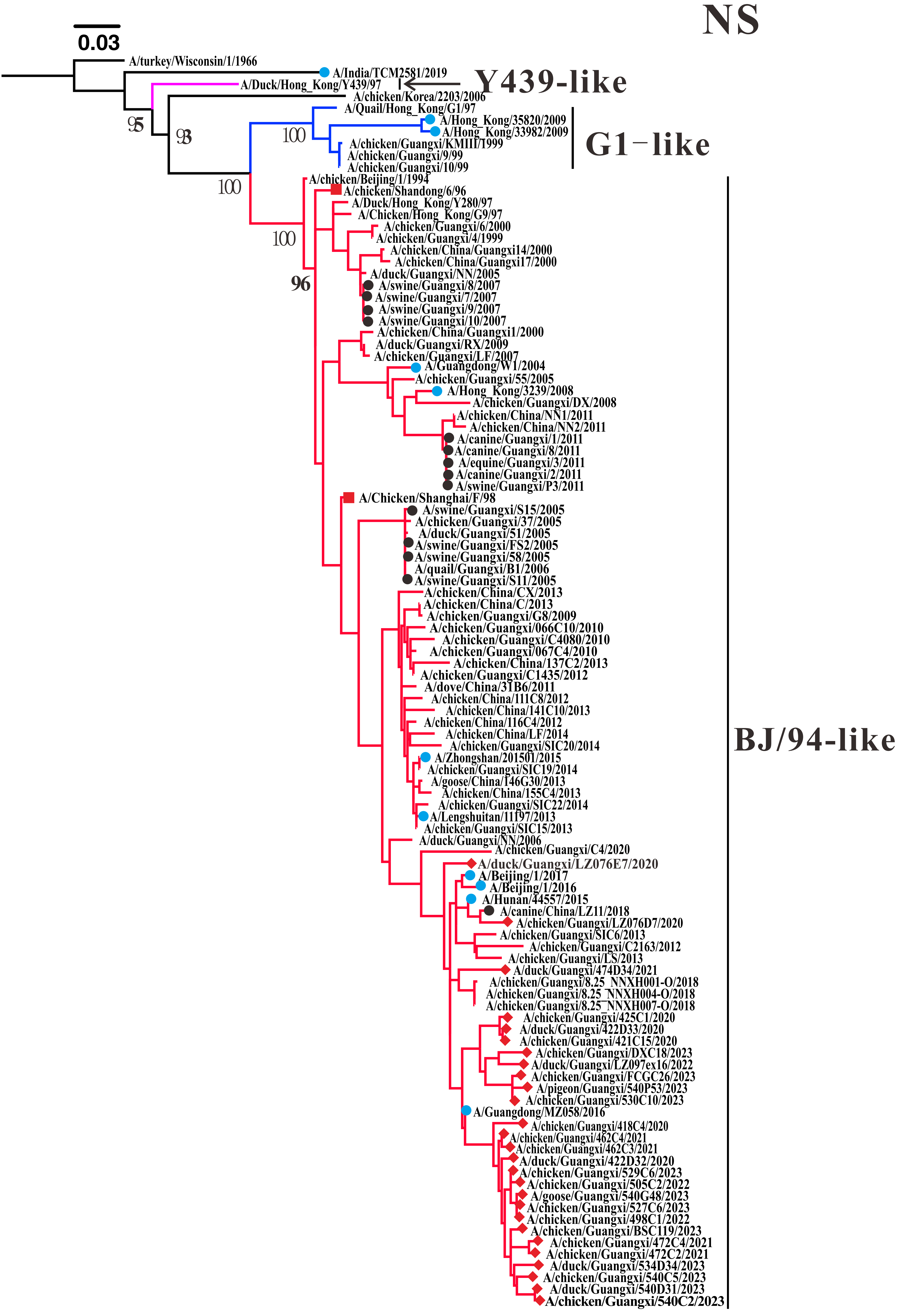

Figure S1. ML trees of the HA, NA, PB2, PB1, PA, NP, M, and NS genes of H9N2 AIV
