# Supplementary material for: Genetic Evolution of H9N2 Avian Influenza Virus in Guangxi, China
Source: Microorganisms. 2025 Nov 12;13(11):2579. doi: 10.3390/microorganisms13112579 (PMC12654625; doi:10.3390/microorganisms13112579)
Supplement: Supplementary file 1 [file microorganisms-13-02579-s001.zip › Figure S2.pdf]

Figure S2

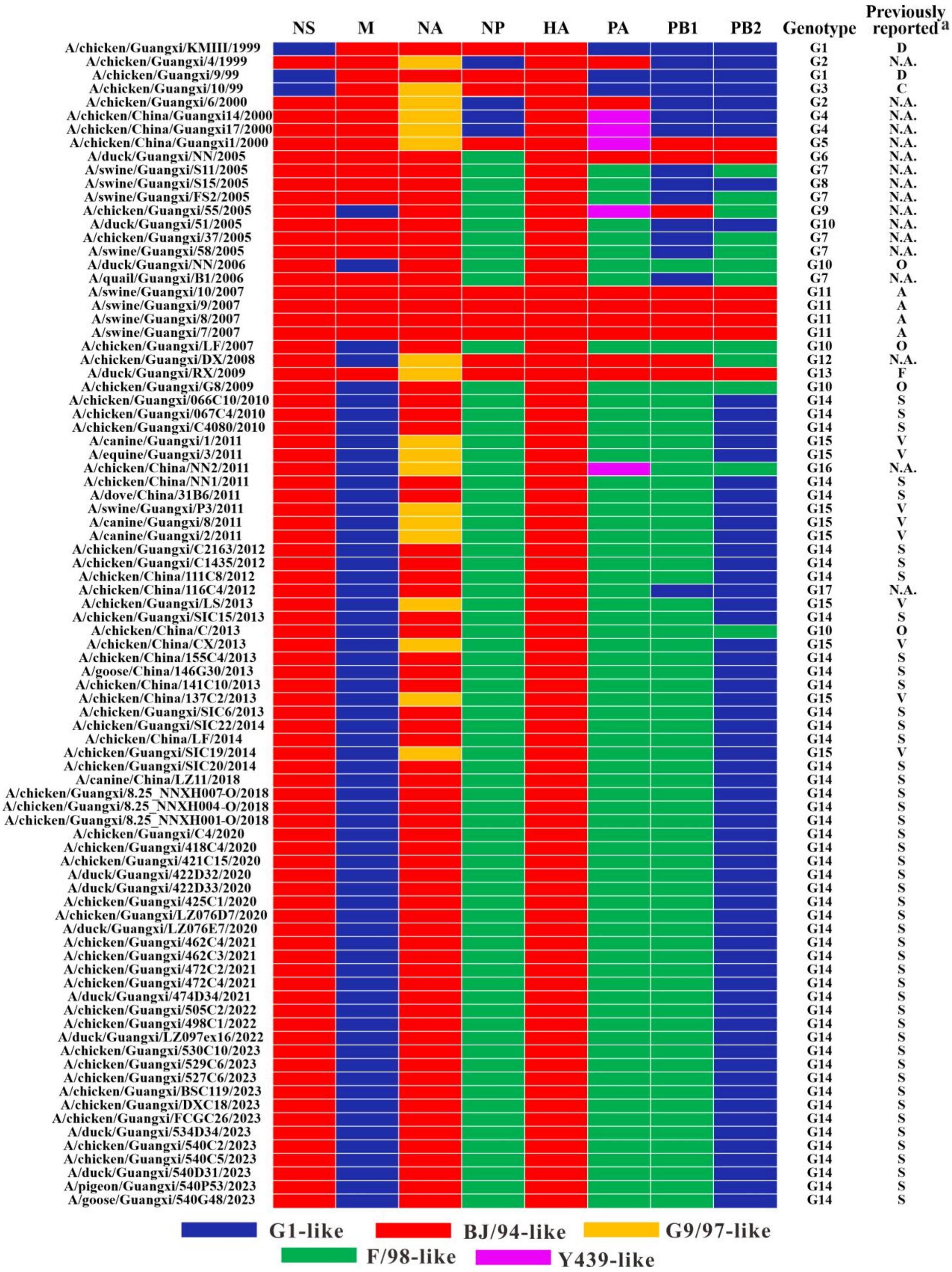

Figure S2. Genotypes of H9N2 AIVs in Guangxi from 1999–2023

The superscript ‘a’ represents the genotypes described by Gu [28]; N.A.: not available.
