# Supplementary material for: Genetic Evolution of H9N2 Avian Influenza Virus in Guangxi, China
Source: Microorganisms. 2025 Nov 12;13(11):2579. doi: 10.3390/microorganisms13112579 (PMC12654625; doi:10.3390/microorganisms13112579)
Supplement: Supplementary file 1 [file microorganisms-13-02579-s001.zip › Table S3,6-8.pdf]

Table S3: Antigenic site mutation frequency in HA protein of H9N2 AIV from Guangxi, 1999-2023

No.	Position*	References	Conservative or no	Surface or no	Vaccine strains (A/chicken/Shanghai/F/1998, A/Chicken/Guangdong/SS/94, and A/Chicken/Shandong/6/96)	The proportion (%) of amino acid substitutions in the 29 Guangxi H9N2 AIVs from 1999~2010	The proportion (%) of amino acid substitutions in the 29 Guangxi H9N2 AIVs from 2011~2018	The proportion (%) of amino acid substitutions in the 28 Guangxi H9N2 AIVs from 2020~2023
1	90	[32]	Conservative	Surface	G	G(93.1), E(6.8)	G(62.0), E(37.9)	G(7.1), E(92.8)
2	133	[32]	Conservative	No	Q	Q(100)	Q(100)	Q(100)
3	138	[32]	Conservative	No	T	T(96.5), S(3.4)	T(86.2), S(13.7)	T(100)
4	145	[30-31, 33]	No	Surface	S	D(3.4), S(79.3), G(10.3), N(6.8)	D(20.6), S(79.3)	D(100)
5	147	[29, 34]	Conservative	Surface	T	T(100)	T(100)	T(100)
6	149	[29-30]	No	Surface	K	K(100)	K(79.3), N(13.7), T(6.8)	K(7.1), N(67.8), T(21.4), S(3.5)
7	153	[29, 34]	No	Surface	D	D(96.5), S(3.4)	D(79.3), G(20.6)	G(85.7), S(14.2)
8	163	[35]	No	Surface	T	T(96.5), N(3.4)	T(96.5), N(3.4)	T(60.7), N(39.2)
9	164	[34]	No	Surface	Q	Q(96.5), R(3.4)	Q(79.3), R(20.6)	R(100)
10	166	[30, 33, 36]	No	Surface	N/D	D(34.4), N(62.0), T(3.4)	D(34.4), N(65.5)	D(21.4), N(78.5)
11	167	[33-34]	No	Surface	N	N(100)	G(17.2), N(82.7)	G(50), N(50)
12	168	[33-34, 37]	No	Surface	A	A(93.1), N(6.8)	A(62.0), D(6.8), E(3.4), N(27.5)	E(21.4), N(78.5)
13	170	[29]	Conservative	Surface	P	P(100)	P(100)	P(100)
14	180	[31]	No	Surface	R	Q(20.6), R(79.3)	Q(82.7), R(17.2)	Q(96.4), D(3.5)
15	196	[34]	No	Surface	D	D(89.6), E(10.3)	D(96.5), E(3.4)	D(42.8), E(57.1)
16	197	[29, 31]	No	Surface	T	T(96.5), D(3.4)	D(17.2), T(82.7)	D(82.1), T(3.5),S(14.2)

17	198	[32, 33, 38]	No	Surface	A	A(37.9), T(34.4),V(27.5)	A(44.8), T(20.6), V(34.4)	A(3.5), T(57.1), V(39.2)
18	200	[30, 34]	No	Surface	T	K(13.7), R(3.4), T(82.7)	R(17.2), T(82.7)	R(96.4), S(3.5)
19	201	[29-30, 32]	No	No	N	N(100)	G(6.8), N(89.6), T(3.4)	G(17.8), N(14.2), T(60.7), S(3.5), A(3.5)
20	206	[29]	Conservative	No	T	T(100)	T(100)	T(100)
21	207	[34]	Conservative	Surface	D	D(100)	D(100)	D(100)
22	215	[33]	Conservative	Surface	E	E(100)	E(100)	E(100)
23	220	[33, 34, 37]	No	Surface	T	T(96.5), I(3.4)	T(72.4), I(27.5)	V(14.2), I(85.7)
24	230	[30]	Conservative	No	L	L(100)	L(96.5), R(3.4)	L(92.8), R(7.1)
25	234	[29]	Conservative	No	Q	L(48.2), M(3.4), Q(48.2)	L(100)	L(100)
26	235	[31-32]	Conservative	No	Q	Q(100)	Q(44.8), M(51.7), L(3.4)	M(100)
27	252	[31]	Conservative	Surface	R	R(100)	R(100)	R(100)
28	282	[32]	Conservative	No	N	K(61.5), N(34.4), R(3.8)	K(100)	K(100)
29	287	[34]	No	No	V	T(27.5), V(72.4)	A(3.4), T(79.3), V(17.2)	T(85.7), I(14.2)
30	294	[32]	No	No	R	K(27.5), R(72.4)	K(82.7), R(17.2)	K(92.8), R(7.1)

Note: The numbers in parentheses represent the proportion of amino acid substitutions in the total Guangxi H9N2 AIV strains.*: H9 numbering (with signal peptide)

Table S6. Amino acid at positions 234 and 235 (H9 numbering) of 86 H9N2 virus strains isolated in Guangxi in different period

Years	The no. of isolates of amino acid combinations at positions 234 and 235				Years	The no. of isolates of amino acid at position 234	
	QQ	LL	LQ	LM		L	Q
1999~2010	13	0	16	0	1999~2010	16	13
2011~2023	0	1	13	43	2011~2023	57	0
total	13	1	29	43	total	73	13

Table S7. Amino acid combinations at positions 234 and 235 (H9 numbering) of 86 H9N2 virus strains isolated in Guangxi from 1999 to 2023

Positions		The no. of isolates	The percentage of isolates (%)	Years of isolation (no.)
234	235			
Q	Q	13	15.1	1999 (3), 2000 (2), 2005 (2), 2006 (1), 2007 (4), 2009 (1)
L	L	1	1.2	2013 (1)
L	Q	29	33.7	1999 (1), 2000 (2), 2005 (6), 2006 (1), 2007 (1), 2008 (1), 2009 (1), 2010 (3), 2011 (7), 2012 (4), 2013 (2)
L	M	43	50	2011 (1), 2013 (6), 2014 (4), 2018 (4), 2020 (8), 2021 (5), 2022 (3), 2023 (12)

Table S8: Antigenic site mutation frequency in NA protein of H9N2 AIV from Guangxi, 1999-2023

No.	Position*	References	Conservative or no	Vaccine strains (A/chicken/Shanghai/F/1998, A/Chicken/Guangdong/SS/94, and A/Chicken/Shandong/6/96)	The proportion (%) of amino acid substitutions in the 29 Guangxi H9N2 AIVs from 1999~2010	The proportion (%) of amino acid substitutions in the 29 Guangxi H9N2 AIVs from 2011~2018	The proportion (%) of amino acid substitutions in the 28 Guangxi H9N2 AIVs from 2020~2023
1	198	[39]	Conservative	D	D(100)	D(100)	D(100)
2	199	[39]	No	K	K(68.9), R(27.5), N(3.4)	K(62.0), R(34.4), N(3.4)	K(75.0), R(10.7), N(14.2)
3	338	[39]	No	R	R(96.5), K(3.4)	R(58.6), K(41.3)	R(96.4), K(3.5)
4	125	[40]	No	G	S(20.6), G(79.3)	S(65.5), G(31.0), D(3.4)	S(96.4), G(3.5)
5	296	[40]	No	K	K(72.4), R(27.5)	K(68.9), R(31.0)	K(100)
6	248	[40]	Conservative	G	G(100)	G(100)	G(100)
7	253	[40]	Conservative	R	R(100)	R(100)	R(100)
8	344	[40]	No	R	R(96.5), S(3.4)	R(100)	R(42.8), K(53.5), E(3.5)
9	356	[40]	No	N	V(31.0), S(27.5), N(20.6), D(20.6)	D(68.9), S(31.0)	D(100)
10	368	[40]	No	K/E	K(51.7), E(27.5), S(13.7), D(6.8)	K(34.4), S(17.2), N(48.2),	N(100)
11	369	[40]	No	D	D(93.1), N(6.8)	D(65.5), G(34.4)	D(3.5), G(46.4), S(50.0)
12	400	[40]	Conservative	S	S(100)	S(100)	S(100)

Note: The numbers in parentheses represent the proportion of amino acid substitutions in the total Guangxi H9N2 AIV strains.*: H9 numbering (with signal peptide)
